# Variation in rhizosphere microbial communities and its association with the symbiotic efficiency of rhizobia in soybean

**DOI:** 10.1038/s41396-020-0648-9

**Published:** 2020-04-27

**Authors:** Qin Han, Qun Ma, Yong Chen, Bing Tian, Lanxi Xu, Yang Bai, Wenfeng Chen, Xia Li

**Affiliations:** 1 State Key Laboratory of Agricultural Microbiology, College of Plant Science and Technology, Huazhong Agricultural University, No. 1 Shizishan Road, Hongshan District, Wuhan, 430070 Hubei China; 2 State Key Laboratory of Plant Genomics, Institute of Genetics and Developmental Biology, The Innovative Academy of Seed Design, Chinese Academy of Sciences, Beijing, 100101 China; 3 State Key Laboratory of Agrobiotechnology, College of Biological Sciences and Rhizobium Research Center, China Agricultural University, Beijing, 100193 China

**Keywords:** Microbial ecology, Agricultural genetics

## Abstract

Rhizobia–legume symbiosis is an important type of plant–microbe mutualism; however, the establishment of this association is complicated and can be affected by many factors. The soybean rhizosphere has a specific microbial community, yet whether these organisms affect rhizobial nodulation has not been well investigated. Here, we analyzed the compositions and relationships of soybean rhizocompartment microbiota in three types of soil. First, we found that the rhizosphere community composition of soybean varied significantly in different soils, and the association network between rhizobia and other rhizosphere bacteria was examined. Second, we found that some rhizosphere microbes were correlated with the composition of bradyrhizobia and sinorhizobia in nodules. We cultivated 278 candidate *Bacillus* isolates from alkaline soil. Finally, interaction and nodulation assays showed that the *Bacillus cereus* group specifically promotes and suppresses the growth of sinorhizobia and bradyrhizobia, respectively, and alleviates the effects of saline–alkali conditions on the nodulation of sinorhizobia as well as affecting its colonization in nodules. Our findings demonstrate a crucial role of the bacterial microbiota in shaping rhizobia–host interactions in soybean, and provide a framework for improving the symbiotic efficiency of this system of mutualism through the use of synthetic bacterial communities.

## Introduction

Nitrogen is an important element for all organisms; it is derived from dinitrogen (N_2_), which comprises up to 78% of the earth’s atmosphere, via nitrogen fixation. Nitrogen fixation converts N_2_, a metabolically useless form of nitrogen for most organisms, into ammonia, which can be metabolized by most organisms. Thus, nitrogen fixation is essential for life. Nitrogen fixation is carried out mainly in soil by nitrogen-fixing bacteria and archaea [1]. Among nitrogen-fixing bacteria, rhizobia can live in soil as saprophytes or in the root nodules of their host legumes as symbionts. In these nodules, rhizobia fix atmospheric nitrogen for use by their host, while the host supplies the rhizobia with carbon from photosynthesis [2, 3]. This symbiosis between rhizobia and legumes is a supreme example of plant–microbe mutualism, and it is beneficial not only for leguminous plants (e.g., soybean, chickpea, pea, common bean, and alfalfa) but also for the global nitrogen cycle [4].

Underlying rhizobia–legume symbiosis is a complex process consisting of several stages, including rhizobial infection of the legume roots, nodule development, nodule functioning, and nodule senescence [5, 6]. Although the rhizobia–legumes interaction is well recognized, this mutualistic association is highly specific and widely diverse [7, 8]. For instance, *Rhizobium leguminosarum* bv. *trifolii* can infect only clover species (*Trifolium* spp.) [9], whereas *Sinorhizobium fredii* NGR234 exhibits a broad host range and can infect up to 112 legume genera [10]. The selective recognition and infection of the root cells of leguminous hosts by rhizobia is a prerequisite for the successful establishment of symbiosis. For a given host, successful infection by rhizobia depends not only on the competitive ability of different rhizobial species but also the ability of rhizobia to cope with various fluctuating environmental factors, including soil properties and soil pH levels [11–16].

In soybean, *Sinorhizobium* (*Ensifer*) and *Bradyrhizobium* are the two main groups of microsymbionts, and they differ in their nodulation abilities [17, 18]. Remarkably, these rhizobia compete with each other in soils with different pH levels. In acidic soil, *Bradyrhizobium* strains such as *Bradyrhizobium diazoefficiens* USDA110 are predominant in nodules of the soybean cv. Williams; conversely, in alkaline soil, *Sinorhizobium* strains are predominant in soybean nodules [19–21]. It has been proposed that the adaptability of these rhizobia to soils with different pH values underlies the biogeographic patterns of soybean root nodulation mediated by *Bradyrhizobium* and *Sinorhizobium* [22]. Indeed, *Sinorhizobium* species are dominant in alkaline–saline soils, whereas *Bradyrhizobium* are dominant in neutral to acidic soils [23–25].

Soil also contains billions of microorganisms, including bacteria and fungi [26]. Rhizobia may compete with these microorganisms in the soil or rhizosphere of their prospective host legume to establish a symbiotic relationship [27]. Leguminous plants, such as *L. japonicum*, *M. truncatula,* and soybean, reportedly play a crucial role in the establishment of bacterial assemblages in the rhizosphere or root, and the symbiosis between rhizobia and legumes is directly affect the structure of the microbiota in these two compartments [28–32]. A loss of function of genes (e.g., *Nod factor receptor 5*, *Nodule inception*, and *Lotus histidine kinase1*) that comprise the common nodulation signaling pathway or mediate the alteration of flavonoids and nitrate, which affect the rhizobial infection of legumes, can reshape the rhizosphere microbiome [33, 34]. Plants establish intimate relationships with diverse microorganisms forming complex communities which vary across host plants and environments, and alteration of this balance may affect host growth or cause disease [35, 36]. Extensive evidence shows that the root-associated microbiome can influence the outcomes of plant–pathogen interactions [37–41]. Thus, we speculated that rhizobia–legume symbiosis might also be affected by the commensal microbes living in soil or inhabiting plant niches. Moreover, in soybean, certain specific functional groups were more representative in the rhizosphere than in the bulk soil and involved functions including the metabolism of nitrogen, iron, phosphorus, and potassium [30], but whether they affect host–rhizobia interactions has not been established.

In this study, to understand the interactions between soybean plants, rhizobia, and the local microbiota, we examined the compositions and network relationships of rhizocompartment microbiota in three types of soil. In addition, we identified the microorganisms associated with rhizobial nodulation, isolated candidate strains, and finally explored their roles in rhizobial nodulation. Our findings provide the first evidence of a role for native microbiota in the adaptation of rhizobia to their environment and in the modulation of the symbiotic efficiency of rhizobia.

## Materials and methods

### Soil types and rhizobia

Experimental soils were collected at a depth of 15 cm from three major soybean cultivation areas in China in fall 2016: Wuhan (30°48′ N, 114°36′ E), Hubei Province; Luancheng (37°94′ N, 114°72′ E), Hebei Province; and Siping (43°51′ N, 124°81′ E), Heilongjiang Province (Supplementary Fig. S1). The soils, which were not cultivated with soybean or were covered with grasses and weeds, were stored in a box at ambient temperature. The physicochemical properties of the samples were analyzed at the Wuhan Academy of Agricultural Sciences Testing Center (Supplementary Table S1). The samples represent three types of soil: acidic (Ac), alkaline (Al), and neutral (Ne), respectively, and the soils had different nutrient element contents. The microbial composition and diversity were determined under the following soil conditions: (I) natural, untreated soil (Ac, Ne, and Al); (II) heat*-*treated soil (treated three times at 80 °C for 20 min; HAc, HNe, and HAl); (III) acidic or neutral soil amended with 50% (w/w) alkaline soil (Ac/Al and Ne/Al); and (IV) acidic or neutral soil amended with lime (final pH 8.2) (Fig. 3a).

Members of the *Bradyrhizobium* genus (*B. diazoefficiens* USDA110, *B.* *elkanii* USDA76, and *B. japonicum* 15781) and *Sinorhizobium* genus (*S. fredii* CCBAU45436, J18-31, and HH103) were used. Strains USDA110, USDA76, 15781, J18-3, and CCBAU45436 were provided by China Agricultural University; strain HH103 was obtained from Huazhong Agricultural University. All strains were grown for 3–4 days at 28 °C on tryptone yeast (TY) medium, pelleted by centrifugation (4500 rpm for 10 min), and suspended in distilled water.

### Greenhouse experiments

Chlorine gas-sterilized soybean (*Glycine max* cv. Williams 82) seeds were grown separately in pots (10 × 10 cm) containing the above soil. Each pot was sown with six soybean seeds and placed in a greenhouse under long-day conditions (16-h photoperiod, 20 °C/28 °C, night/day). After 5 days, the seedlings were thinned to 3–4 pot^−1^, and the plants were watered with tap water as needed. Bulk soil, rhizosphere, root, and nodule samples were collected at 28 days as described by Bulgarelli [42] and Xiao [31]. Unplanted soil samples were used as controls (bulk soil). For each treatment, there were four replicates of 3–4 seedlings each.

### 16S rRNA gene sample preparation, sequencing, and analysis

In total, 48 samples (12 bulk soil, 12 rhizosphere, 12 root, and 12 nodule samples) from untreated soil and 56 samples (28 rhizosphere and 28 nodule samples) from treated soil were used for sequencing (Supplementary Table S2). Microbial DNA was extracted using an E.Z.N.A.® Soil DNA Kit (Omega Bio-Tek Inc., Norcross, GA, USA). The V5–V7 hypervariable regions of the bacterial 16S rRNA gene were amplified using primers 799F [43] and 1193R [44]. PCR was performed as follows: 3 min at 95 °C; 27 cycles of 30 s at 95 °C, 30 s at 55 °C, and 45 s at 72 °C; and 72 °C for 10 min. The reactions were run in triplicate 20-μL mixtures containing 4 μL of 5× FastPfu Buffer, 2 μL of 2.5 mM dNTPs, 0.8 μL of each primer (5 μM), 0.4 μL of FastPfu Polymerase, and 10 ng of template DNA. The products were extracted from 2% agarose gels, purified using an AxyPrep DNA Gel Extraction Kit (Axygen Biosciences, Union City, CA, USA), and quantified using QuantiFluor™-ST (Promega, Madison, WI, USA) according to the manufacturers’ protocols.

The amplicons were pooled in an equimolar concentration and paired-end sequenced (2 × 300) on an Illumina MiSeq platform (Illumina, San Diego, CA, USA) according to standard protocols at Majorbio Bio-Pharm Technology Co. Ltd (Shanghai, China). Operational taxonomic units (OTUs) were clustered with a 97% similarity cutoff using UPARSE (version 7.1; http://drive5.com/uparse/); chimeric sequences were identified and removed using UCHIME. The taxonomy of each 16S rRNA sequence was analyzed using the RDP Classifier algorithm (http://rdp.cme.msu.edu/) against the Silva (SSU123) 16S rRNA database with a confidence threshold of 70%. The alpha-diversity indexes of Chao1 and Shannon were calculated by Mothur software [45]. A network analysis was performed by using Networkx software [46]. Only Spearman correlations with an *r* > 0.6 (*P* < 0.05) were considered to indicate a valid interactive event. A principal coordinates analysis (PCoA) based on Bray–Curtis distances was performed using the R package vegan (version 2.1). Linear discriminant analysis coupled with an effect size measurements (LEfSe) analysis was conducted to search for significantly different (*P* < 0.05) taxa between two groups, with an LDA score of at least 3.5 [47].

### Microbe isolation and identification

To isolate putative *Bacillus* from alkaline soil, 1 g of soil was resuspended in sterile phosphate buffer, incubated for 30 min with shaking at 150 rpm, and then the suspension was heat-treated at 80 °C for 20 min and spiral plated on Luria-Bertani (LB) medium [48]. One representative of each colony type as determined by its morphology was selected, purified, and stored at −80 °C in LB medium containing 20% glycerol until further use. The classifications of the isolates at the genus level were confirmed by 16S rRNA gene sequencing. Draft genome sequencing was performed by Shanghai Majorbio Biopharm Technology Co., Ltd (Shanghai, China), using an Illumina HiSeq 2000 system. The genome sequences were used to query the NCBI database to obtain nearest*-*neighbor sequences and the genomic distances of the sequenced isolates were compared by JSpeciesWS (http://jspecies.ribohost.com/jspeciesws/#home).

### Interaction assay

Candidate strains were inoculated in 5 mL of LB medium and incubated overnight at 28 °C with shaking at 180 rpm. The OD of the bacterial cultures was adjusted to 0.5 at 600 nm. Strains USDA110 and CCBAU45436 were grown for 3–4 days at 28 °C on TY medium, and the OD readings of the cultures were adjusted to 1.0 and 0.5, respectively, at 600 nm. Next, an isolate or 1.5 μL suspension of each candidate strain was spotted at a 1.2- or 0.6-cm distance from *B. diazoefficiens* USDA110 or *S. fredii* CCBAU45436 on YMA plates. The plates were incubated at 28 °C for 4–6 days. Images were captured using a Leica microscope (DFC495). Each assay was carried out in four replicates.

### Nodulation assay

Chlorine gas-sterilized soybeans were planted in pots (10 × 10 cm) containing sterilized vermiculite and grown for 4 days. The plants were transplanted to fresh vermiculite with and without 25 mM NaHCO_3_ and 75 mM NaCl, respectively. The plants were then inoculated with 20 mL suspension of *B. diazoefficiens* USDA110 or *S. fredii* CCBAU45436 (OD600 = 0.1) and simultaneously treated with 30 mL sterile water (the control) or a *Bacillus* suspension (OD600 = 0.5). The plants were watered with a weak nitrogen nutrient solution (pH 7) as needed. After 28 days of inoculation and treatments, the numbers of nodules per plant were determined. Each treatment included four replicates of two seedlings each. The experiments were repeated twice under identical conditions.

For the colonization assays, soybean plants were grown in vermiculite under similar conditions as those described above. The plants were coinoculated with 20 ml of mixed rhizobia suspensions (USDA110:USDA76:15781:CCBAU45436:J18–3: HH103 = 1:1:1:1:1:1, OD600 = 0.1) and treated (or not) with 30 mL of a mixed *Bacillus* (three strains) suspension (OD600 = 0.5). Plants were watered with a weak nitrogen nutrient solution (pH 7) as needed. Twenty-eight days after inoculation, the nodules were collected, and the *Bradyrhizobium* and *Sinorhizobium* in the nodules were quantified as described by Trabelsi [49] with some modifications. In brief, total DNA from the 500 mg bacteroids from each treatment was extracted using an E.Z.N.A.® Soil DNA Kit (Omega Bio-Tek Inc., Norcross, GA, USA). The overall quality and quantity of each DNA sample were examined with BioPhotometer D30 (Eppendorf). All quantitative PCRs were carried out in 96-well plates using a Bio-Rad CFX Connect Real-Time system (Bio-Rad, USA). Genus-specific primer sets were used to analyze the *nodC* and *mlr6601* [50] genes in *Bradyrhizobium* and *Sinorhizobium*, respectively (Supplementary Table S3). Standard curves were obtained using serial dilutions of CCBAU45436 or USDA110 genomic DNA. The populations of *Bradyrhizobium* and *Sinorhizobium* were calculated from the cycle threshold values linear regression coefficients derived from the standards of each strain and adjusted to gene copies per μL bacteroid DNA solution. Each treatment included four replicates of two seedlings each. The experiments were repeated twice under identical conditions.

### Statistical analysis

Graphical representations were generated with GraphPad Prism 5 (GraphPad Software, Inc., La Jolla, CA, USA). The means and standard deviations of the data were calculated. Kruskal–Wallis *H* test was utilized to identify taxa significantly different among the soil types at phylum or family level. A paired Wilcoxon rank-sum test was performed to compare the alpha diversity of different soil types. Permutational multivariate analysis of variance (PERMANOVA) was carried out to measure effect sizes and significance differences in beta diversity. Comparisons of *Bacillus* and H_2_O treatments or Mix-R and Mix-R + *Bacillus* treatments were performed by nonparametric Mann–Whitney tests (GraphPad Prism).

## Results

### Composition and diversity of the soybean rhizocompartment microbiota in different types of soils

To investigate the microbial composition and diversity of soybeans grown in different soils, we collected three different types of soil from three main soybean production areas including Wuhan (Hubei Province), Siping (Heilongjiang Province), and Luancheng (Hebei Province), and the pH levels of these soils were 5.63 (acidic), 7.2 (neutral), and 8.23 (alkaline), respectively (Supplementary Table S1). Accordingly, we collected root, rhizosphere, nodule, and bulk soil samples as described by Bulgarelli et al. [42] and Xiao et al. [31] (Supplementary Fig. S1). The V5–V7 regions of the 16S rRNA gene were amplified by PCR and sequenced on an Illumina MiSeq platform. In total, 1,797,926 high-quality, nonchimeric sequences were yielded with a median sequence per sample value of 37,457 (range 30,032–44,608) from 48 samples (Table S4). The rarefaction curves of compartment samples based on the OTU numbers are shown in Supplementary Fig. S2a. Sequencing data were rarefied to the lowest number of reads observed in a single sample and 3107 bacterial OTUs were identified. General features of the high-throughput sequencing results as well as taxon numbers at all levels are shown in Supplementary Table S5. The Good’s coverage for the observed OTUs was 98.65 ± 0.08% (mean ± s.e.m.), and except between bulk soil and rhizosphere samples, there were significant differences in the Chao1 index values between the other compartment samples (Supplementary Fig. S2b).

**Fig. 1:**
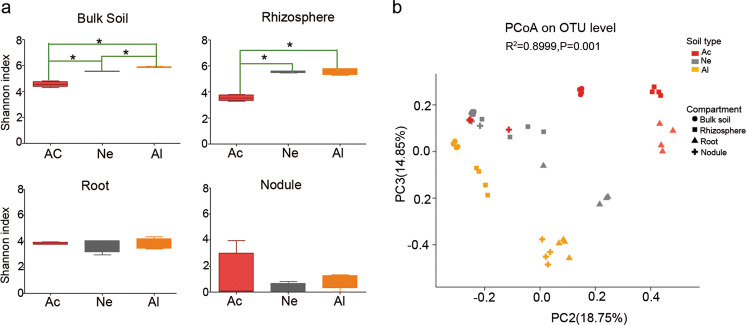
Alpha and beta-diversity of the soybean rhizocompartment microbiota in three types of soils. **a** Alpha-diversity (Shannon indexes) measurements for microbial communities from the acidic soil (Ac), neutral soil (Ne), and alkaline soil (Al) by compartments. Statistical analyses were performed by paired Wilcoxon rank-sum test, and significance is denoted by asterisks where *P < 0.05. Data are presented as median values ± SDs (*n* = 4). **b** PCoA of Bray–Curtis distances reveals that soil type is a major source of bacterial community variation in both the rhizosphere and roots. *N* = 108. Clustering significance by soil type was determined by Adonis (Pr(>F) = 0.001). Each point corresponds to a different sample colored by soil type, and each compartment is represented by a different shape.

For the bulk soil, the bacterial community composition varied significantly in the different soils (Supplementary Fig. S3). The relative abundances of major microbial phyla, including Acidobacteria, Proteobacteria, and Chloroflexi were significantly higher in acidic soil than those in neutral (Ne) or alkaline (Al) soils, whereas Actinobacteria, Firmicutes, and Gemmatimonadetes were more abundant in neutral (Ne) and alkaline (Al) soils than in acidic (Ac) soil (false discovery rate (FDR) adjusted *P* < 0.05, Kruskal–Wallis *H* test) (Supplementary Fig. S4a). The differences in Proteobacteria and Actinobacteria in the three types of soils were even greater in the rhizosphere samples (Supplementary Fig. S4b) but were not observed in root (Supplementary Fig. S4c) and nodule (Supplementary Fig. S4d) samples. The differences in the bacterial composition of the rhizocompartments in different soil types were also observed at the family level, and the trend was consistent with that at the phylum level (Supplementary Fig. S5). In the top ten families, all species in bulk soil samples (Supplementary Fig. S5a), six species in rhizosphere samples (Supplementary Fig. S5b), five species in root samples (Supplementary Fig. S5c), and only two species in nodule samples were significantly different, and belonged to *Rhizobiaceae* and *Bradyrhizobiaceae* (Supplementary Fig. S5d). Alpha-diversity analysis (Shannon index) showed that the differences among the soybean root and nodule microbial communities in three types of soils were less pronounced in the tested soils than in the rhizosphere or bulk soil samples (Fig. 1a). A PCoA of Bray–Curtis distances (beta diversity) revealed that the bulk soil (circular), rhizosphere (square), and root (triangle) microbiota in three types of soil exhibited a clear separation (Fig. 1b and Supplementary Fig. S6a). For the nodule compartment, the acid (Ac) and neutral (Ne) soil samples were clustered together but well separated from the alkaline (Al) soil samples (Supplementary Fig. S6b). PERMANOVA based on the Bray–Curtis (Supplementary Table S6) and weighted UniFrac (Supplementary Table S7) distances confirmed that the microbial communities in the three rhizocompartments of soybean were all significantly different (*P* < 0.01) when grown in the three types of soils.

### Microbial cooccurrence and interaction networks in the different rhizocompartments

**Fig. 2:**
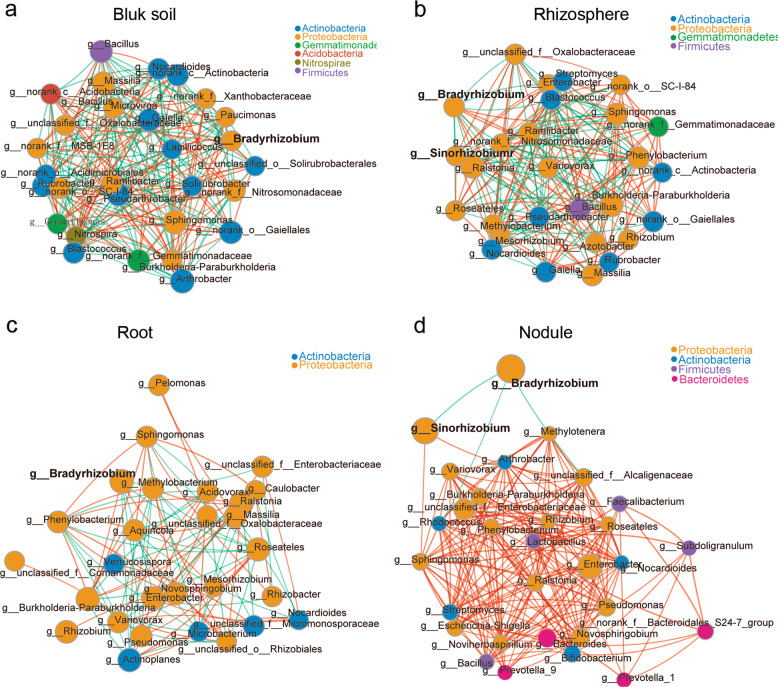
Microbial interaction networks in the different compartments. The interaction network of dominant microbiota at the genus level (top 30) in the bulk soil (**a**), rhizosphere (**b**), roots (**c**), and nodules (**d**). The size of the nodes shows the abundance of OTUs, and the different colors indicate the corresponding taxonomic assignment at the phylum level. The edge color represents positive (red) and negative (green) correlations. The edge thickness indicates the correlation values; only significant interactions are shown (*r* > 0.6; *P* < 0.05).

The interaction between different microbial strains is one of the main driving factors of population structure and dynamics, because microbes can cooccur or exclude each other [51, 52]. Hence, we next used Networkx software to analyze the interaction networks in different compartments. The Spearman correlation values between genera were calculated based on their occurrence patterns across samples from the three different soil types. Our results showed a high level of node connectivity within the rhizocompartment microbiota. In an analysis of the top 30 bacteria at the genus level, there were 202 correlations in the bulk soil (Fig. 2a and Supplementary Table S8), 184 correlations in the rhizosphere (Fig. 2b and Supplementary Table S9), 90 correlations in the root (Fig. 2c and Supplementary Table S10), and 185 correlations in the nodule (Fig. 2d and Supplementary Table S11) samples. These results suggest that the interaction networks in the rhizosphere and root are relatively simple compared with that in bulk soil. In nodules, the correlation among bacteria increased while microbial network complexity declined. With the exception of *Bradyrhizobium* being negatively correlated with *Sinorhizobium* (−0.847637), *Rhodococcus* (−0.623598), and *unclassified_f__Alcaligenaceae* (−0.770069), the connectivity indicated that all of the other genera were positively correlated (Fig. 2d), suggesting that these taxa can cooccur with rhizobium in soybean nodules and will not exclude each other. In addition, we found that the negative correlation between *Bradyrhizobium* and *Sinorhizobium* (−0.70403) was also observed in rhizosphere samples. Furthermore, *Bradyrhizobium* and *Sinorhizobium* were correlated (positive and negative) with 14 and 13 other different rhizospheric genera, respectively (Fig. 2b and Supplementary Table S9), which may affect the nodulation of these two types of rhizobia.

### The composition of rhizobia in soybean nodules is soil condition-dependent

**Fig. 3:**
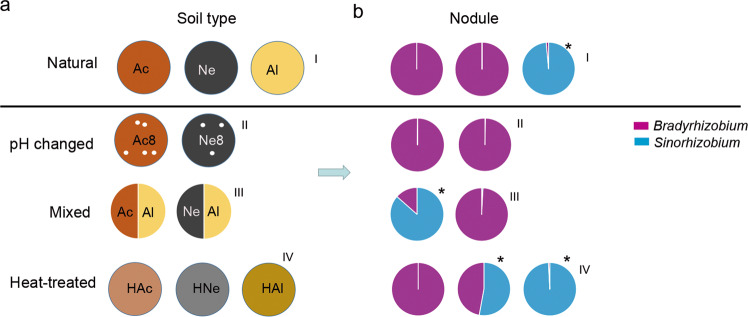
Relationship between soil treatment and composition of rhizobia in the rhizosphere and nodules. **a** Schematic diagram of the soil treatments. Acidic soil (Ac), neutral soil (Ne), alkaline soil (Al), acidic soil plus lime (Ac8), neutral soil plus lime (Ne8), heated acidic soil (HAc), neutral soil (HNe), alkaline soil (HAl), alkaline soil amended with 50% (w/w) acidic soil (Ac/Al) or neutral soil (Ne/Al). **b** Relative abundance of *Bradyrhizobium* and *Sinorhizobium* in nodules of plants planted in the soil shown in **a**. I, II, III, and IV represent normal, pH changed, mixed, and heat-treated soils, respectively. Purple represents *Bradyrhizobium*. Blue represents *Sinorhizobium*. Asterisks show that the relative abundance of *Sinorhizobium* was higher than that of *Bradyrhizobium*.

Among the natural acidic or neutral-soil nodule communities (Fig. 3a–I), *Bradyrhizobium* was the most abundant genus, making up ~99.97% of the total abundance; by contrast *Sinorhizobium* was dominant in the nodules from the alkaline soil (98.56%) but very rare in the acidic (0.081%) or neutral-soil nodules (0.007%) (Fig. 3b–I). To further explore the influence of soil factors on the composition of rhizobia in the nodules, we conducted artificial interference on the planted soil. First, we changed the pH of the acidic or neutral soil by supplementing with lime (pH 8.2) (Fig. 3a–II); second, we mixed acidic or neutral soil with alkaline soil (1:1; w/w) (Fig. 3a–III); last, we heated the soil at 80 °C for 20 min (Fig. 3a–IV) before plant cultivation. Sterilized soybean seeds were grown in these soils for 28 days, and the rhizosphere soils (28 samples) and nodules (28 samples) were collected as described above. Amplification and sequencing of these 56 samples yielded 2,703,009 high-quality sequences (average of 48,268 and range of 30,585–95,823 sequences per sample) (Supplementary Table S12). Rarefaction curves of the samples from the two compartments based on the OTU numbers are shown in Supplementary Fig. S7. The sequences were clustered into OTUs using the same criteria as those described above, yielding 2830 microbial OTUs and the Chao1 and Shannon indexes of the two compartments were significantly different (FDR adjusted *P* < 0.001, Wilcoxon rank-sum test) (Supplementary Table S13).

Upon alteration of the pH of acidic or neutral soils to create alkaline conditions, the abundance of *Sinorhizobium* in root nodules was not increased (Fig. 3b–II and Supplementary Table S14), implying that, in addition to soil pH, other factors in the soils may also affect the composition of rhizobia in nodules. When soybean was grown in the acidic–alkaline mixed soil (pH 6.93), the relative nodule abundance of *Sinorhizobium* (86.56%) exceeded that of *Bradyrhizobium*, while in neutral–alkaline mixed soil (pH 7.71), the nodule abundance of *Sinorhizobium* was not significantly changed (Fig. 3b–III and Supplementary Table S14). Further heat-treated experiments showed that, in heat-treated neutral soil, the relative abundance of *Sinorhizobium* in nodules was significantly increased (54.64%, Supplementary Table S14). However, this change was not found in soybean plants grown in heat-treated acidic soil (Fig. 3b–IV). These results together suggested that the effect of planted soil on the composition of rhizobia in nodules was complex and not dependent on the soil pH alone.

### Rhizosphere microbiomes were associated with nodulation of rhizobia

**Fig. 4:**
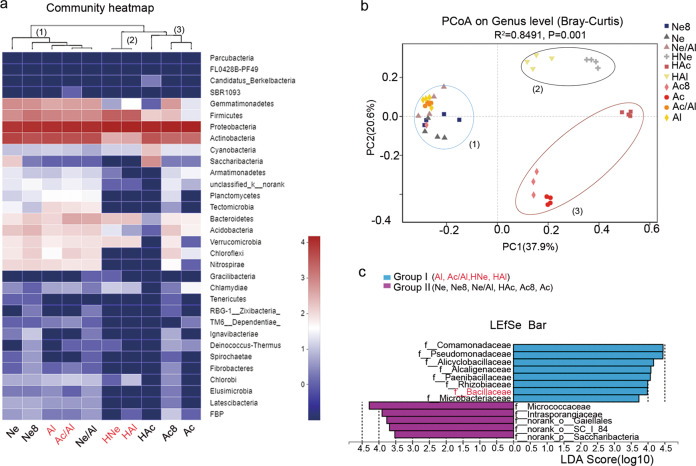
The rhizosphere microbial community structure was associated with the nodulation of *Bradyrhizobium* and *Sinorhizobium*. **a** Composition and clustering of bacterial microorganisms from different rhizosphere samples on the phylum level. **b** PCoA analysis based on Bray–Curtis distances of the rhizosphere microbiomes of soybean seedlings grown in soils shown in Fig. 3a; *n* = 40. Clustering significance was determined by Adonis (Pr(>F) = 0.001). Acidic soil (Ac), neutral soil (Ne), alkaline soil(Al), acidic soil plus lime (Ac8) and neutral soil plus lime (Ne8), alkaline soil amended with 50% (w/w) acidic soil (Ac/Al) or neutral soil (Ne/Al) and heated acidic soil (HAc), neutral soil (HNe), alkaline soil (HAl). **c** Linear dis**c**riminant analysis (LDA) coupled with the effect size measurements identifies the significant abundance of data in **b**. Taxa enriched in Group I (purple) and Group II (blue) are indicated with LDA scores, respectively. Only taxa with LDA values greater than 3.5 (*P* < 0.05) are shown.

To determine whether the change in the composition of rhizobia in the nodules was related to the rhizospheric bacteria, we analyzed the microbial community composition of ten rhizosphere samples and conducted a cluster analysis. The hierarchical clustering results of the heatmap analysis divided the ten rhizosphere samples into three main clusters based on the dominant phyla, and the three clusters hosted distinct bacterial assemblages at the phylum level (Fig. 4a). These clusters were confirmed by a PCoA based on the Bray–Curtis distances and PERMANOVA (*P* < 0.05) (Supplementary Table S15), and obvious clustering among the ten soil treatments was observed. The primary axis of variation (explaining 37.9% of the overall variation) separated cluster 3 (Ac, Ac8, and HAc) and cluster 2 (HNe and HAl) from cluster 1 (Ne, Ne8, Al, Ac/Al, Ne/Al, HNe, and HAl), while the secondary axis of variation (explaining 20.6% of the overall variation) distinguished cluster 2 from cluster 3 (Fig. 4b).

Next, we divided these ten treatments into groups I (Al, Ac/Al, HNe, and HAl) and group II (Ne, Ne8, Ne/Al, Ac, HAc, and Ac8) according to the composition of rhizobia in the nodules (Fig. 3b) and utilized the linear discriminant analysis effect size (LEfSe) algorithm. The results showed that eight and five families (LDA log score threshold >3.5 and *P* < 0.05) most likely explained the differences between groups I and II, respectively. *Comamonadaceae* showed the highest LDA score (5.16) in group I, followed by *Pseudomonadaceae* (4.89), *Alicyclobacillaceae* (4.09), *Paenibacillaceae* (4.7), *Rhizobiaceae* (3.99), *Bacillaceae* (3.98), and *Microbacteriaceae* (3.73), which were identified as the dominant key families in group I and may be related to *Sinorhizobium* nodulation. In comparison, indicator bacteria in group II clustered into the families *Micrococcaceae* (4.29), *Intrasporangiaceae* (3.90), *norank_o__Gaiellales* (3.77), *norank_o__SC_I_84* (3.68), and *norank_p__Saccharibacteria* (3.52), which may be involved in *Bradyrhizobium* nodulation.

### Differential effects of *Bacillus* on the growth of rhizobia in vitro

Among the above indicated families, *Bacillaceae* are beneficial microorganisms in plant–microbe interactions that have been detected in many plants rhizospheres and are well-known for their plant disease resistance- and plant growth-promoting properties [53–55]. The facts that *Bacillaceae* (4.30% of OTUs in group I) was present in high abundance in alkaline rhizosphere samples (Supplementary Fig. S5b) and *Bacillus* showed a significant positive correlation (*r* = 0.714537) to *Sinorhizobium* in rhizosphere samples (Fig. 2b and Supplementary Table S9) promoted us to explore a possible mechanistic role of *Bacillus* in rhizobial nodulation. To this end, we isolated the candidate *Bacillus* strains from alkaline soil by heat treatment. First, 278 candidate *Bacillus* isolates were selected and their interactions with *S. fredii* CCBAU45436 were examined on YMA medium (0.7% agar). The results showed that a group of isolates (~12.6%), which had similar morphology, significantly promoted the growth of *S. fredii* CCBAU45436. The rest of the tested isolates showed little or no effect on the growth of CCBAU45436 (Supplementary Fig. S8). Three representative isolates (B-9, B-11, and B-13) with obvious promoting effects were identified as the *Bacillus cereus* group (belonging to OTU1511) by 16S rRNA-based methods (Supplementary Table S16). To further identify these strains on the species level, we conducted genome sequencing by Illumina Hiseq platform. Based on their average nucleotide identity (ANI) values, the three strains were reclassified as *Bacillus albus* B-9 (95.61% ANI to *B. albus* N35–10–2), *B. cereus* B-11 (97.98% ANI to *B. cereus* ATCC 14579), and *B. albus* B-13 (98.26% ANI to *B. albus* N35–10–2) (Supplementary Table S17).

**Fig. 5:**
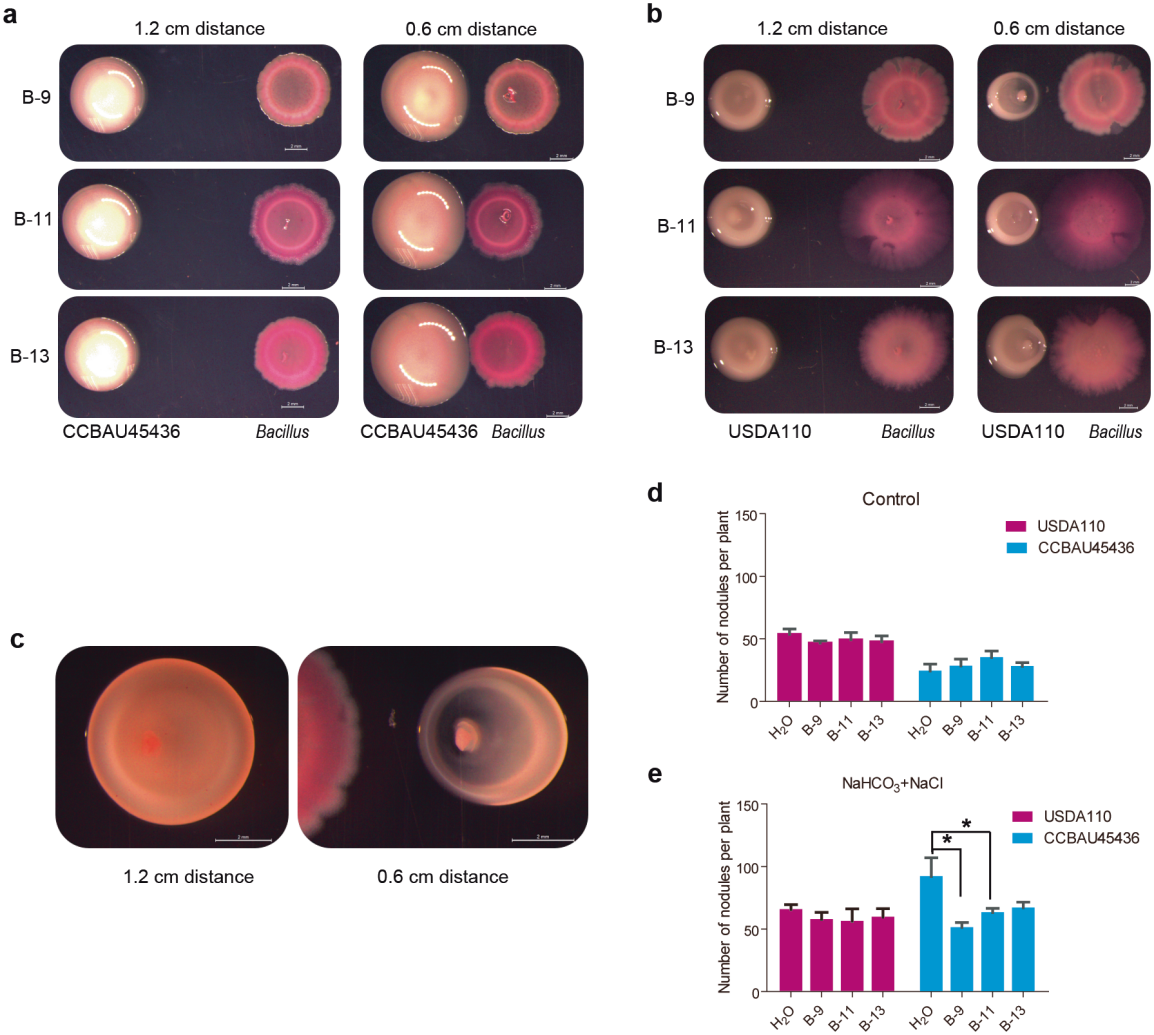
Effect of *Bacillus* on the growth and nodulation of rhizobia. **a** *Bacillus* colonies were inoculated next to a *S. fredii* CCBAU45436 colony at distances of 1.2 cm or 0.6 cm on a YMA plate. **b** *Bacillus* colonies were inoculated next to a *B. diazoefficiens* USDA110 colony at distances of 1.2 cm or 0.6 cm. **c** The growth phenotype of B-13 after contact with *S. fredii* CCBAU45436 in **a**. **d** The reverse colony of *B. diazoefficiens* USDA110 inoculated next to *Bacillus* B-9 in **a**. The results represent one of three replicates with similar results. The scale bar represents 2 mm. **e** The nodule numbers of CCBAU45436- or USDA110-inoculated plants treated with H_2_O or *Bacillus* under control conditions. **f** The nodule numbers of CCBAU45436- or USDA110-inoculated plants treated H_2_O or *Bacillus* under saline–alkali conditions (25 mM NaHCO_3_ + 75 mM NaCl); the experiment was repeated twice. Statistical analyses were performed by Mann–Whitney nonparametric tests and significance is denoted by asterisks where * indicates *P* < 0.05. Data are presented as median value ± SD (*n* = 4).

When CCBAU45436 and three *Bacillus* strains were cocultured at different distances (0.6 or 1.2 cm) on the YMA medium for 4 days, the colony diameters (1.14 cm) of *S. fredii* CCBAU45436 at a 0.6 cm distance (to *Bacillus*) were significantly greater than those of colonies (0.91 cm) located at the 1.2 cm distance (Fig. 5a, *n* = 12, *P* < 0.0001, nonparametric Mann–Whitney tests). More interestingly, when *B. diazoefficiens* USDA110 and *Bacillus* were cocultured on YMA medium, the growth of USDA110 adjacent to *Bacillus* was markedly inhibited (Fig. 5b), becoming elliptical (Fig. 5c). The obvious growth promotion of CCBAU45436 and inhibition of USDA110 were not observed with *Pseudomonas* strains (also isolated from alkaline soil) (Supplementary Fig. S9). In addition, *Bacillus* also stimulated *S. fredii* CCBAU45436 growth but inhibited *B. diazoefficiens* USDA110 growth in the gamma-radiation-sterilized alkaline soil (Supplementary Fig. S10). Collectively, these results indicated that the *B. cereus* group may specifically promote and inhibit the growth of CCBAU45436 and USDA110, respectively, once again indicating an interaction of the *Bacillus* genus with rhizobia.

### *Bacillus* alleviates the effect of saline–alkali conditions on the nodulation phenotype of CCBAU45436 in the greenhouse

Next, we examined the effect of *Bacillus* on the nodulation ability of CCBAU45436 and USDA110 in the greenhouse, and added different concentrations of sodium percarbonate and sodium chloride to vermiculite to simulate salt–alkali conditions [56]. Under control conditions (pH 7), the nodule number of soybeans inoculated with USDA110 (63) was more than that in plants inoculated with CCBAU45436 (54), but the difference was not significant. With the increase in pH (concentrations of bicarbonate ions), the nodule number in both rhizobia-inoculated plants increased, but the nodule number of plants inoculated with CCBAU45436 increased more obviously than that under the control conditions (Supplementary Fig. S11c), and the highest nodule number (165) was associated with smaller size (Supplementary Fig. S11a) and leaf chlorosis (Supplementary Fig. S10b) at pH 8 (25 mM NaHCO_3_ + 75 mM NaCl). For the USDA110-inoculated plants, the number of nodules was highest (128) at pH 8.5 (50 mM NaHCO_3_ + 50 mM NaCl) (Supplementary Fig. S11c), and the chlorosis was less severe than that of CCBAU45436-inoculated plants (Supplementary Fig. S11b).

*Bacillus* treatment did not significantly affect the nodule numbers of plants inoculated with either CCBAU45436 or USDA110 in pH 7 (control) conditions (Fig. 5d); in sharp contrast, *Bacillus* treatment (B-9 and B-11) restored the nodule phenotypes (reduced nodule number) of the CCBAU45436-inoculated plants, whereas *Bacillus* did not affect the nodule number or size of the USDA110-inoculated plants under pH 8 (25 mM NaHCO_3_ + 75 mM NaCl) conditions (Fig. 5e). Taken together, these results indicate that CCBAU45436-soybean symbiosis is more sensitive to pH than that of USDA110, and *Bacillus* can alleviate the inhibitory effect of pH on CCBAU45436-soybean symbiosis.

### *Bacillus* affects colonization efficiency of *Sinorhizobium* in nodules

**Fig. 6:**
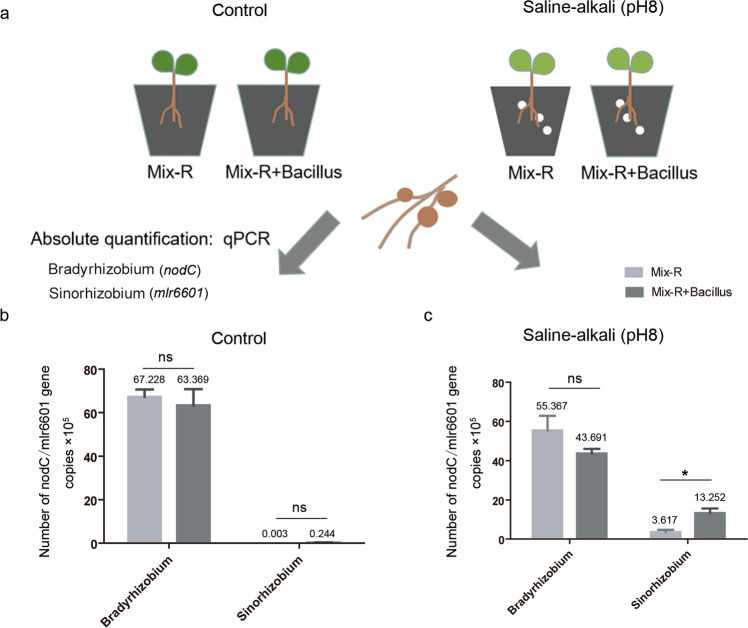
Effect of *Bacillus* on the colonization of rhizobia in nodules. **a** Schematic representation of the mixed inoculation experiment, showing the planting, inoculation, and quantitative detection methods. Three-day-old plants were transplanted into control or saline–alkali-treated vermiculite, and then plants were inoculated with mixed rhizobia or mixed rhizobia containing *Bacillus*. Twenty-eight days later, the bacteroid DNA of the nodules was extracted, the concentrations of two kinds of rhizobia were quantified by qPCR, and the experiment was repeated twice. **b** The populations of *Sinorhizobium* or *Bradyrhizobium* bacteroids under control conditions. **c** The populations of *Sinorhizobium* or *Bradyrhizobium* bacteroids under saline–alkali conditions. Mix-R indicates six strains of rhizobium. qPCR results of the Mix-R and Mix-R + *Bacillus* treatments were analyzed with nonparametric Mann–Whitney tests (*n* = 4, ns nonsignificant; *P < 0.05). The horizontal bars of each graph indicate the median values and are listed where appropriate for clarity.

To further investigate the role of *Bacillus* in the colonization of *Bradyrhizobium* and *Sinorhizobium* in soybean nodules, we conducted mixed rhizobial inoculation experiments. Three *Bradyrhizobium* and three *Sinorhizobium* strains were coinoculated onto soybean plants with or without *Bacillus* under saline–alkali (pH 8) conditions (Fig. 6a). *Sinorhizobium* and *Bradyrhizobium* were more resistant to alkali and acid conditions, respectively (Supplementary Fig. S12a), which is consistent with previous studies [57]. At 28 days after inoculation, the populations of *Sinorhizobium* or *Bradyrhizobium* in the nodules were expressed as the gene copies of *mlr6601* or *nodC*, respectively, that were quantified by qPCR using genus**-**specific primers (Supplementary Table S3 and Fig. S12b). Their respective standard curves were obtained using serial dilutions of CCBAU45436 and USDA110 genomic DNA (Supplementary Fig. S12c). Similarly, there were no significant differences in the populations of *Sinorhizobium* or *Bradyrhizobium* (gene copies) in nodules with and without *Bacillus* treatment under the control conditions (Fig. 6b). After saline–alkali treatment, the gene copies of *mlr6601* (*Sinorhizobium*) in nodules were slightly increased (0.224 up to 3.617 × 10^5^), but significantly increased (up to 13.252 × 10^5^) after coinoculated with *Bacillus* (Fig. 6b, c). The gene copies of *nodC* (*Bradyrhizobium*) before and after inoculation with *Bacillus* were also decreased, but the difference was not significant (Fig. 6c). In addition, we amplified the marker genes of *Bacillus* in nodule samples with the specific primers (OPL-114F-lipo and OPL-114R-lipo) [58], and no bands were detected (data not shown), suggesting that the inoculated *Bacillus* did not enter the nodules. Together, these results show that *Bacillus* may indirectly promote the colonization of *Sinorhizobium* in nodules in a manner dependent on alkali conditions.

## Discussion

Successful symbiosis is regulated by both rhizobia and their legume hosts; moreover, the nodulation rate of rhizobia in a given host is variable and is affected by the environmental factors and symbiotic rhizobia. A well-known example of this is the substantial differences in nodulation rates of *Sinorhizobium* and *Bradyrhizobium* in soybean grown in soils with different pH values; the differences in the pH tolerance of *Sinorhizobium* and *Bradyrhizobium* may explain the geographic distribution patterns of these rhizobia [15, 19, 22, 23]. Comparative genomic analysis revealed that genus-specific genes, known to be involved in alkaline–saline adaptations, likely contribute to the observed biogeographic patterns of *Bradyrhizobium* and *Sinorhizobium* nodulation in soybean [24]. Here, we found that the soybean rhizosphere microbiota, especially the *B. cereus* group, affect the growth and nodulation of *Sinorhizobium* and *Bradyrhizobium*, which may also affect the nodulation of these two kinds of rhizobia.

Previous studies have shown that legumes have a core rhizosphere microbiome whose composition depends on the genotype of the host [30, 59–61]. We found that soybean plants grown in three types of soil have greater microbial diversity in the rhizosphere than in roots (Fig. 2a), which is similar to the results in soybean and alfalfa [31]. These results suggest that the microbial community in roots is more stable in response to a fluctuating growth environment than that in the rhizosphere or soil [62, 63] and that legumes, during evolution, acquired the ability to recruit certain microbes that may be beneficial for their growth. In addition, we found clear correlations in rhizocompartment microorganisms, especially in the rhizosphere, including the interaction between rhizobia and other rhizosphere taxa (Fig. 2b), which is in agreement with the study showing that bacterial subnetworks in both bulk soil and the rhizosphere were most influenced by soil pH in 51 soybean fields across China [64]. Microbial networks reflect cooccurrence patterns and interactions among microorganisms, which may affect the composition of the microbial communities or the interaction between microorganisms and host plants.

Root nodules are the organs that house rhizobia and are the site of symbiotic nitrogen fixation. A previous study showed that the composition of nodule endophytes is plant species-specific; *Sinorhizobium* were the dominant rhizobia in alfalfa, while *Sinorhizobium* and *Bradyrhizobium* were the most abundant genera in soybean nodules [31]. We found that *Sinorhizobium* was the dominant species in nodules in alkaline soil, while *Bradyrhizobium* was dominant in nodules in neutral and acidic soils (Fig. 3d). These results are consistent with previous reports showing that soybean rhizobial communities exhibit strong biogeographical patterns, which are shaped by local climatic and edaphic factors (available iron and soil pH) [23, 24, 65]. In addition, we found that the rhizobial composition in nodules also may be influenced by some other rhizosphere microbiota, such as *Bacillaceae*, which may be involved in the colonization of nodules by *Sinorhizobium* and *Bradyrhizobium* (Fig. 5a, b).

*Bacillaceae* are well-known beneficial rhizosphere and endophytic bacteria and dominate the nonrhizobial subcommunity of the soybean microbiota [64, 66]. Several *Bacillus* strains were reported to affect soybean nodulation [67, 68], however, the role of *Bacillus* in soybean nodulation remains unclear. Intriguingly, our data show that *B. cereus* group strains isolated from the saline–alkaline soil specifically promoted the growth of CCBAU45436 but inhibited the growth of USDA110 (Fig. 5a, b and Supplementary Fig. S10), and we speculated that *Bacillus* may also affect the distribution of rhizobia in soil. Through simulation of saline–alkali conditions with sodium bicarbonate and sodium chloride, we found that with the increase in pH, the nodule number in CCBAU45436-inoculated plants also increased even though the nodules were small, which may also partially explain why *Sinorhizobium* were dominant under alkaline soil conditions. Similarly, under low Pi conditions, some rhizobia can also induce more and smaller nodules, because they are relatively more sensitive to stress [69]. Under normal conditions, *Bacillus* did not affect the nodule number of either CCBAU45436- or USDA110-inoculated plants. However, *Bacillus* treatment restored the nodulation defects (mainly nodule number) of CCBAU45436-inoculated soybean plants under saline–alkali conditions (Fig. 5e) and affected *Sinorhizobium* colonization in nodules as well (Fig. 6). It has been widely reported that root-associated bacteria can alleviate the adverse effects of salinity and alkalinity stress on plants [70, 71], thus it is likely that these root-associated bacteria may be beneficial to the nodulation of those stress sensitive rhizobia in legumes. Cooperative and competitive microbial interactions are important selective forces driving complex microbial assemblages in different compartments for plant fitness [72]. To the best of our knowledge, this study is the first to report the interactive relationship between *Bacillus* and rhizobia and the effect of *Bacillus* on rhizobia–legume symbiotic nodulation under stress. Whether these interactions can occur in the rhizosphere and how *Bacillus* affects the nodulation phenotype and rhizobium colonization under saline–alkali conditions are not yet known. Further investigation of this specific interaction between *Bacillus* and rhizobia will help us to decipher the molecular mechanism by which *Bacillus* regulates rhizobial nodulation and colonization in soybean nodules under different soil conditions.

In summary, our findings demonstrate that the rhizosphere microbiota has an important regulatory role in rhizobia–soybean symbioses and the adaptation of plants to a stress environment. These findings provide new insights into the distribution of soybean-nodulating rhizobia in the field and provide new approaches to promote nodulation of rhizobia under stress conditions in soybean or other leguminous plants. The hologenomes of legumes and their microbial counterpart(s) should be considered as an important component of the genetic basis of rhizobia–legume symbioses and in genetic engineering to increase the efficiency of symbiotic nitrogen fixation.

## Supplementary information

Supplementary Figure legends

Supplementary Table legends

Supplementary Fig S1

Supplementary Fig S2

Supplementary Fig S3

Supplementary Fig S4

Supplementary Fig S5

Supplementary Fig S6

Supplementary Fig S7

Supplementary Fig S8

Supplementary Fig S9

Supplementary Fig S10

Supplementary Fig S11

Supplementary Fig S12

Supplementary Fig S13
