# Supplementary material for: Variation in rhizosphere microbial communities and its association with the symbiotic efficiency of rhizobia in soybean
Source: ISME J. 2020 Apr 27;14(8):1915–28. doi: 10.1038/s41396-020-0648-9 (PMC7367843; doi:10.1038/s41396-020-0648-9)
Supplement: Supplementary file 1 — Supplementary Figure legends [file 41396_2020_648_MOESM1_ESM.doc]

**Supplementary Figure legends**

**Figure S1.** The flow chart of the experimental design including soil type, rhizocompartments and primers for sequencing. Color indicates the sampling sites. Ac, Ne and Al represent acidic, neutral and alkaline soil, respectively.

**Figure S2.** The sampling depths and alpha diversity analysis of samples. **a** Rarefaction curves of soil, rhizosphere, root, and nodule samples from untreated soil. **b** Chao1 indexes of soil, rhizosphere, root, and nodule samples from untreated soil. Statistical analyses were performed by a paired Wilcoxon rank-sum test, and significance is denoted by asterisks where * indicates P < 0.05, (n = 12).

**Figure S3.** The structural composition of the soybean rhizocompartment microbiota in different types of soils at Phylum level.

**Figure S4.** The different relative abundances of maior Phyla (top 10) among the different compartments. **a** bulk soil; **b** rhizosphere; **c** root; **d** nodule. Kruskal-Walis H test was used to evaluate the significance of differences between the indicated groups (n = 4, * indicates P < 0.05; ** indicates P < 0.01; *** indicates P < 0.001).

**Figure S5.** The different relative abundances of major families (top 10) among the different compartments. **a** bulk soil; **b** rhizosphere; **c** root; **d** nodule. Kruskal-Walis H test was used to evaluate the significance of differences between the indicated groups (n = 4, * indicates P < 0.05; ** indicates P < 0.01; *** indicates P < 0.001).

**Figure S6.** PCoA analyses based on Bray-Curtis distance. **a** PCoA analysis based on Bray-Curtis distances of the bulk soil, rhizosphere and root microbiomes of soybean seedlings grown in different soils. n=36. **b** PCoA analysis based on Bray-Curtis distances of the nodules microbiomes of soybean seedlings grown in different soils. n=12. Clustering significance by soil type was determined by Adonis [Pr (>F) = 0.001or 0.003]. Each point corresponds to a different sample colored by soil type, and each compartment is represented by a different shape.

**Figure S7.** Rarefaction curves of rhizosphere and nodule samples from treated soil.

**Figure S8.** Effect of different candidates of *Bacillus* on the growth of *S. fredii* CCBAU45436 on YMA plate. Triangles represent obvious promotion. The square represents slight promotion. Circle means no effect.

**Figure S9.** The effect of *Pseudomonas* on the growth of rhizobia on YMA plate. **a** *Pseudomonas* colonies were inoculated next to a *S. fredii* CCBAU45436 colony at distances of 1.2 cm or 0.6 cm on a YMA plate. **b** *Pseudomonas* colonies were inoculated next to a *B. diazoefficiens* USDA110 colony at distances of 1.2 cm or 0.6 cm. The results represent one of three replicates with similar results. Scale bar represents 2 mm.

**Figure S10.** The effect of *Bacillus* on the growth of rhizobia in alkaline soil. Five grams of gamma-radiation sterilized soil in a 15-mL plastic pipe were inoculated with 1 mL of mixed rhizobia (USDA110:CCBAU45436=1:1, OD600=0.1) and treated with 1 mL of water or Bacillus suspension (OD600=0.5). The numbers of USDA110 and CCBAU45436 cells in the soil were evaluated at 15 days after treatment. In each treatment, soil was resuspended in 10 mL of sterile water and cultured for 30 min at 150 rpm. The suspensions were then serially diluted with sterile water and plated onto YMA containing Spe or Na and Tmp. The number of colonies was calculated after incubation at 28°C for 4–6 days. Significant differences between control and *Bacillus* treatments were measured by nonparametric Mann-Whitney tests (n=4, * indicates P < 0.05).

**Figure S11.** Effect of different pH conditions on nodulation of *B. diazoefficiens* USDA110 and *S. fredii* CCBAU45436. Photographs of soybean root (**a**) and leaf (**b**) phenotypes of plants inoculated with either CCBAU45436 or USDA110 in different pH conditions. **c** The nodule number of plants inoculated with either CCBAU45436 or USDA110 in different pH conditions. pH 7.0 (Control), pH 7.5 (10 mM NaHCO_3_ + 90 mM NaCl), pH 8.0 (25 mM NaHCO_3_ + 75 mM NaCl), pH 8.5 (50 mM NaHCO_3_ + 50 mM NaCl). Significant differences between pH conditions and the two symbiotic associations were measured by Duncan’s multiple range test (n=4, * indicates P < 0.05). The experiment was repeated twice.

**Figure S12.** Growth phenotypes and specific primers of six rhizobia. **a** The growth curves of six rhizobia strains under acidic, neutral and alakaline growth conditions in minimal media (n=4). **b** Gel electrophoresis picture of six rhizobia strains amplified by the genus-specific qPCR primers. Lane 1, 2, and 3 were CCBAU45436, J18-31 and HH103, respectively. Lane 4, 5, and 6 were USDA110, USDA76, and 15781, respectively. **c** Calibration plots on USDA110 and CCBAU45436 DNA for *nodC* and *mlr6601* (n=4), respectively. The threshold cycle (Ct) values with respect to the amounts of USDA110 or CCBAU45436 DNA templates are reported. Equations and linear correlation coefficients (R^2^) are shown.
