# Supplementary material for: Variation in rhizosphere microbial communities and its association with the symbiotic efficiency of rhizobia in soybean
Source: ISME J. 2020 Apr 27;14(8):1915–28. doi: 10.1038/s41396-020-0648-9 (PMC7367843; doi:10.1038/s41396-020-0648-9)
Supplement: Supplementary file 2 — Supplementary Table legends [file 41396_2020_648_MOESM2_ESM.doc]

**Supplementary Table legends**

**Table S1** Soil chemical factors of sampling sites

**Table S2** The number of replicates per sample in the greenhouse

**Table S3** Specific qPCR primers designed for each resident bacterial species

**Table S4** The sequence number of pre treatment in untreated-soil samples

**Table S5** General features of the high-throughput sequencing results in untreated-soil samples

**Table S6** PERMANOVA analysis of the rhizocompartment microbial community in different soil type composition based on Bray-Curtis

**Table S7** PERMANOVA analysis of the soybean microbial community in different soil type composition based on weighted UniFrac

**Table S8** Correlation coefficient of microorganisms (genus level) in bulk soil samples

**Table S9** Correlation coefficient of microorganisms (genus level) in rhizosphere samples

**Table S10** Correlation coefficient of microorganisms (genus level) in root samples

**Table S11** Correlation coefficient of microorganisms (genus level) in nodule samples

**Table S12** The sequence number of pre treatment in treated-soil samples

**Table S13** General features of the high-throughput sequencing results in treated-soil samples

**Table S14** Comparisons of Ensifer relativeabundance between different soil type in rhizosphere sample

**Table S15** PERMANOVA analysis results using Bray-Curtis as a distance metric for three cluster samples

**Table S16** Identification of Bacillus isolates in alkaline soil

**Table S17** Average nucleotide identity (ANI) of the strains by JSpeciesWS
