# Supplementary figures and images for: Variation in rhizosphere microbial communities and its association with the symbiotic efficiency of rhizobia in soybean
Source: ISME J. 2020 Apr 27;14(8):1915–28. doi: 10.1038/s41396-020-0648-9 (PMC7367843; doi:10.1038/s41396-020-0648-9)

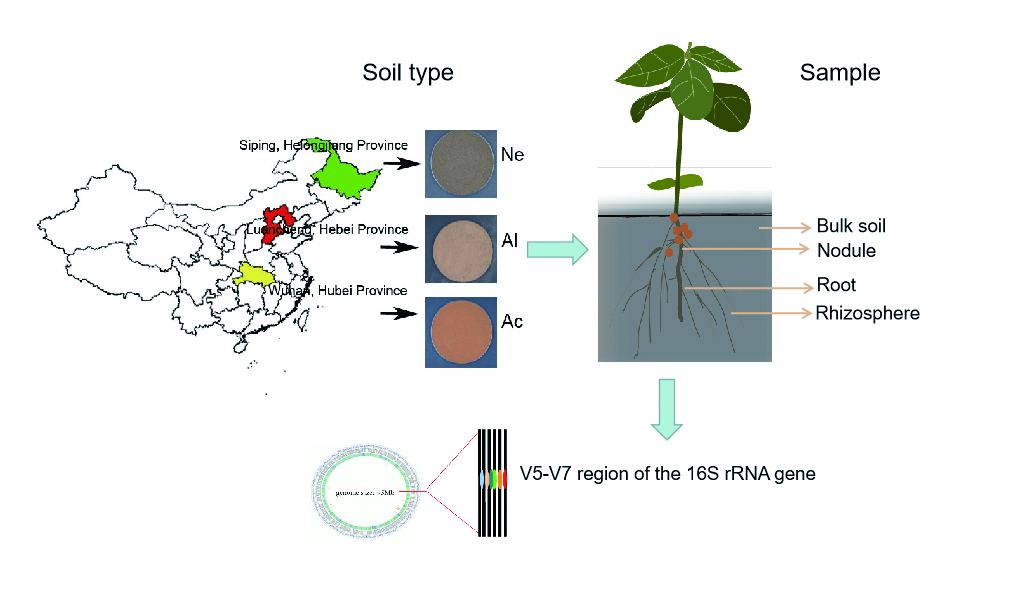

Supplement: Supplementary file 3 — Supplementary Fig S1 [file 41396_2020_648_MOESM3_ESM.tif]

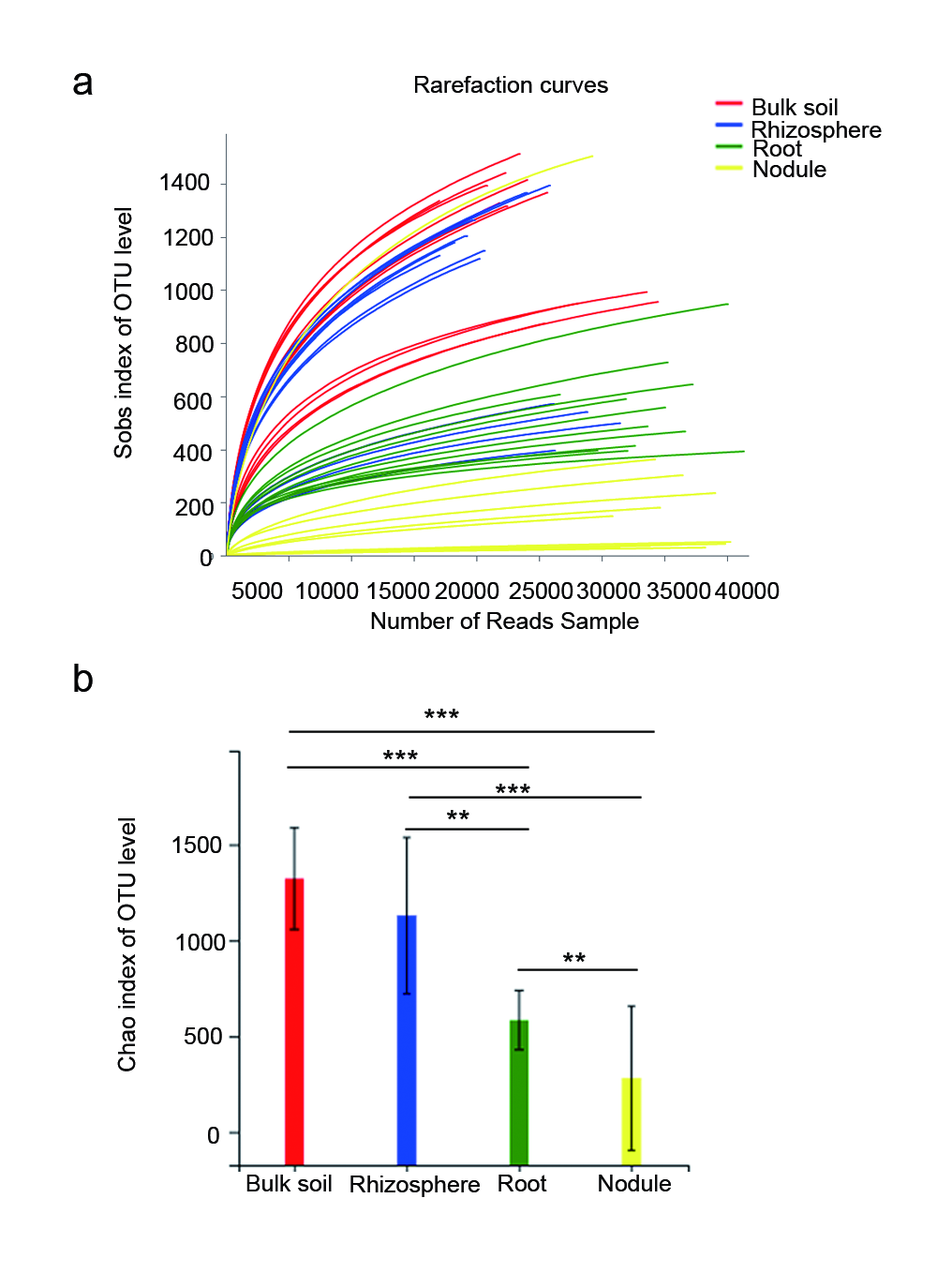

Supplement: Supplementary file 4 — Supplementary Fig S2 [file 41396_2020_648_MOESM4_ESM.tif]

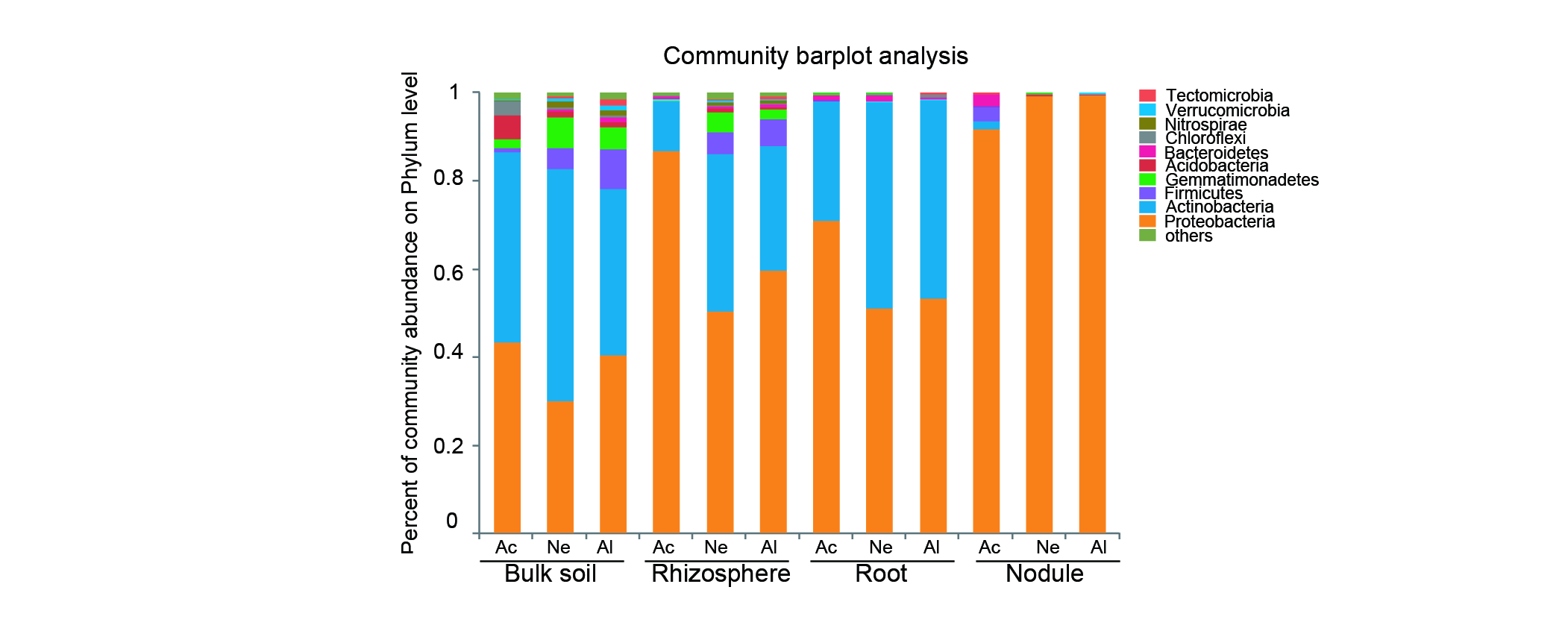

Supplement: Supplementary file 5 — Supplementary Fig S3 [file 41396_2020_648_MOESM5_ESM.tif]

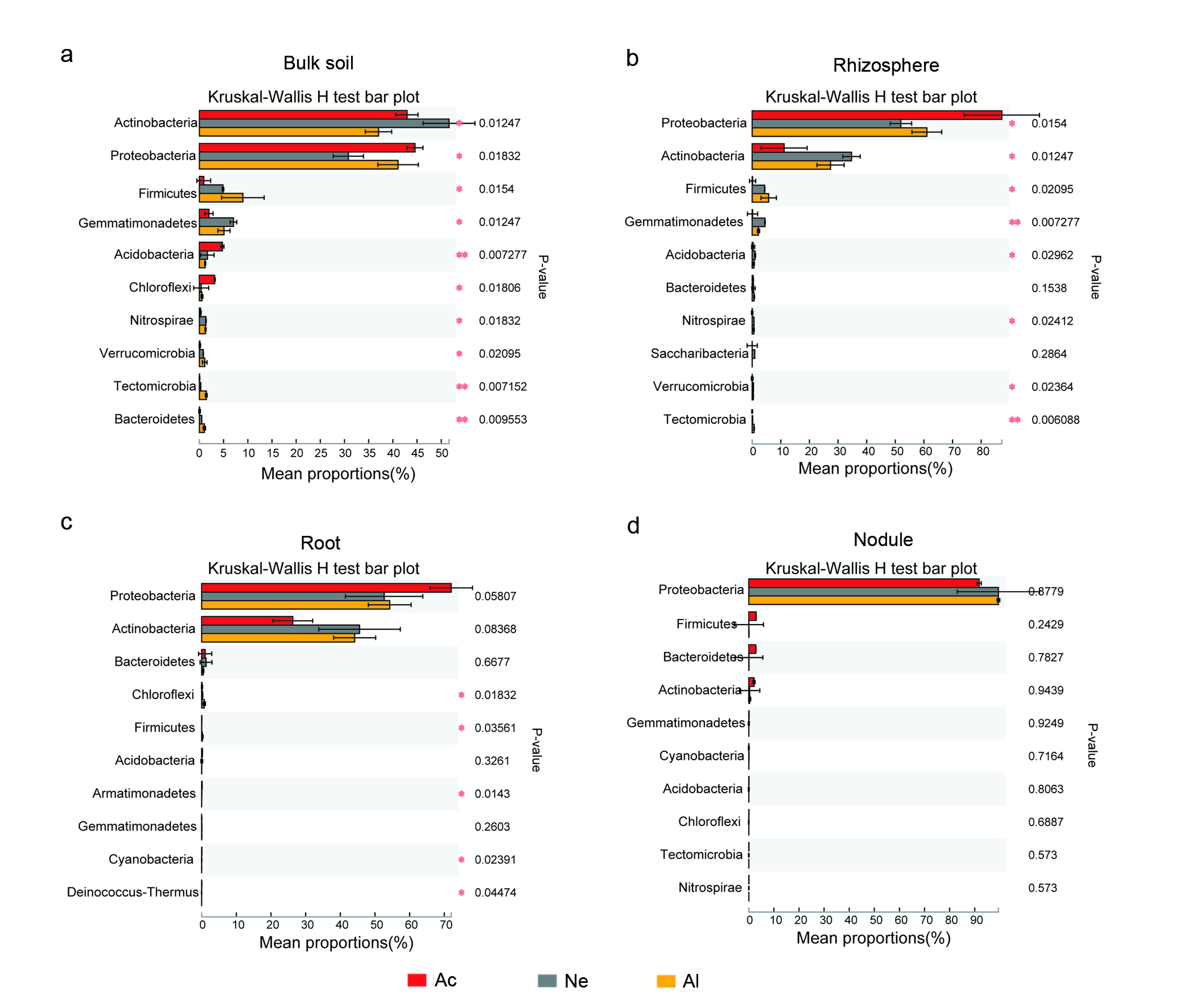

Supplement: Supplementary file 6 — Supplementary Fig S4 [file 41396_2020_648_MOESM6_ESM.tif]

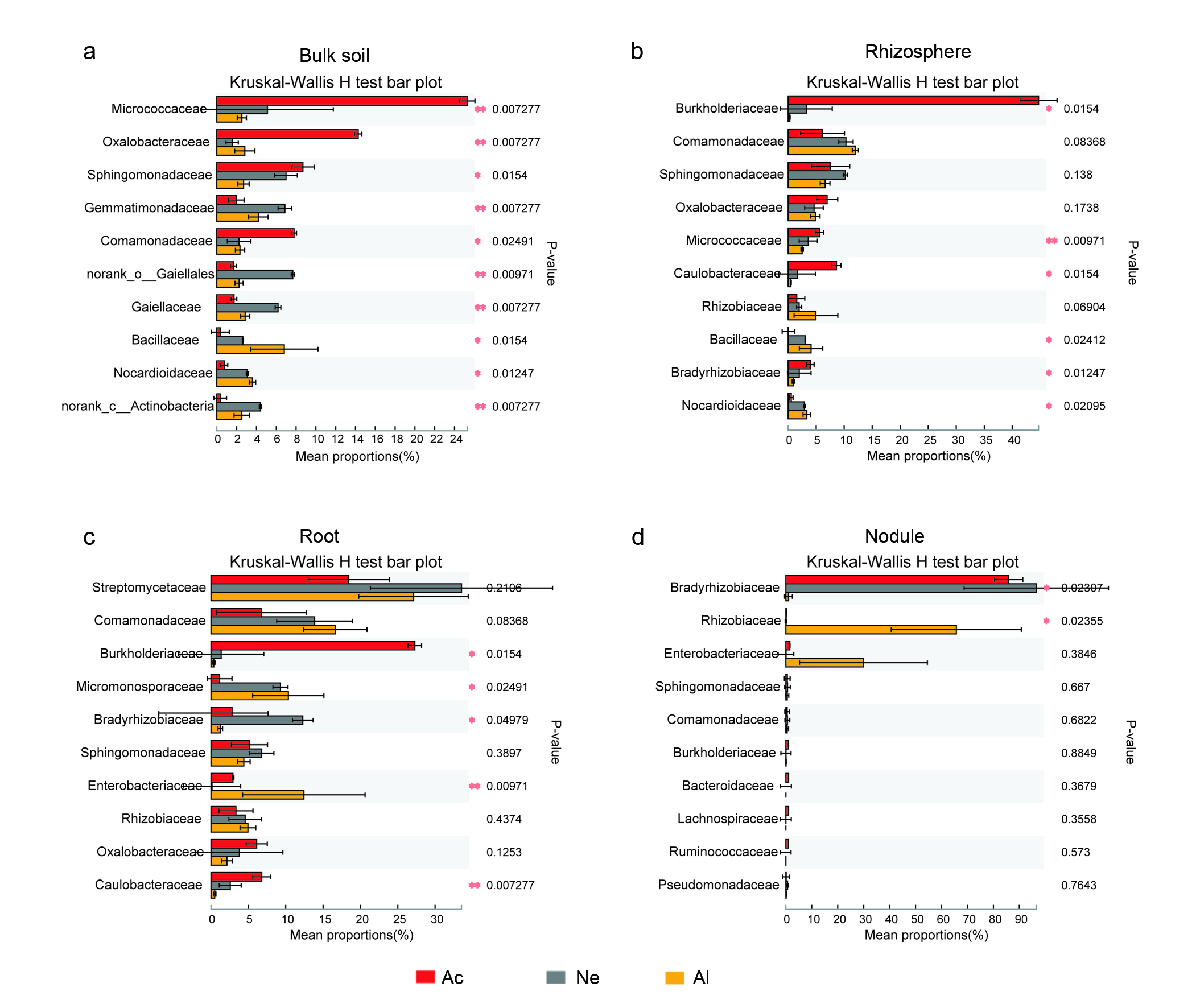

Supplement: Supplementary file 7 — Supplementary Fig S5 [file 41396_2020_648_MOESM7_ESM.tif]

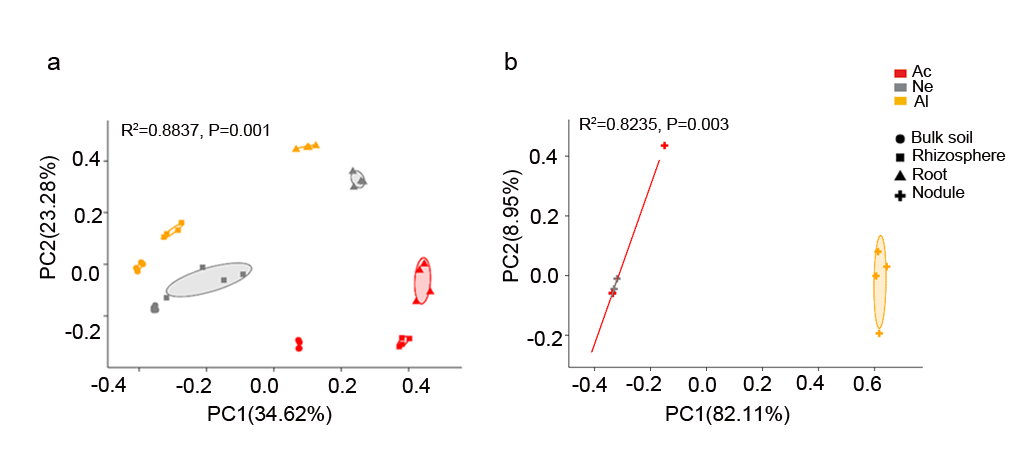

Supplement: Supplementary file 8 — Supplementary Fig S6 [file 41396_2020_648_MOESM8_ESM.tif]

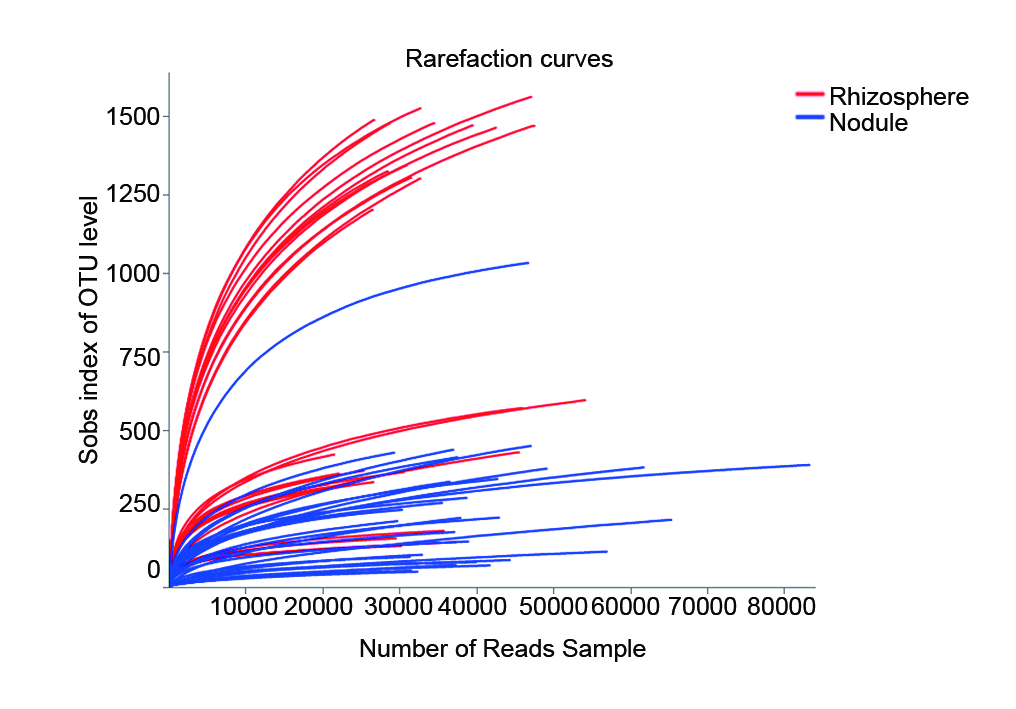

Supplement: Supplementary file 9 — Supplementary Fig S7 [file 41396_2020_648_MOESM9_ESM.tif]

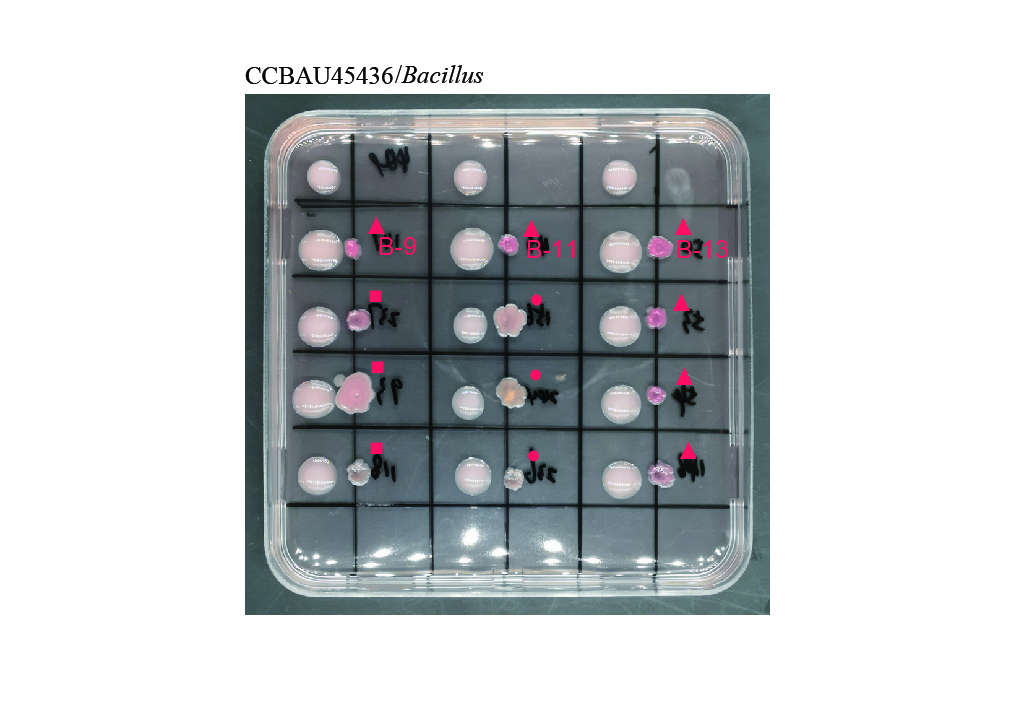

Supplement: Supplementary file 10 — Supplementary Fig S8 [file 41396_2020_648_MOESM10_ESM.tif]

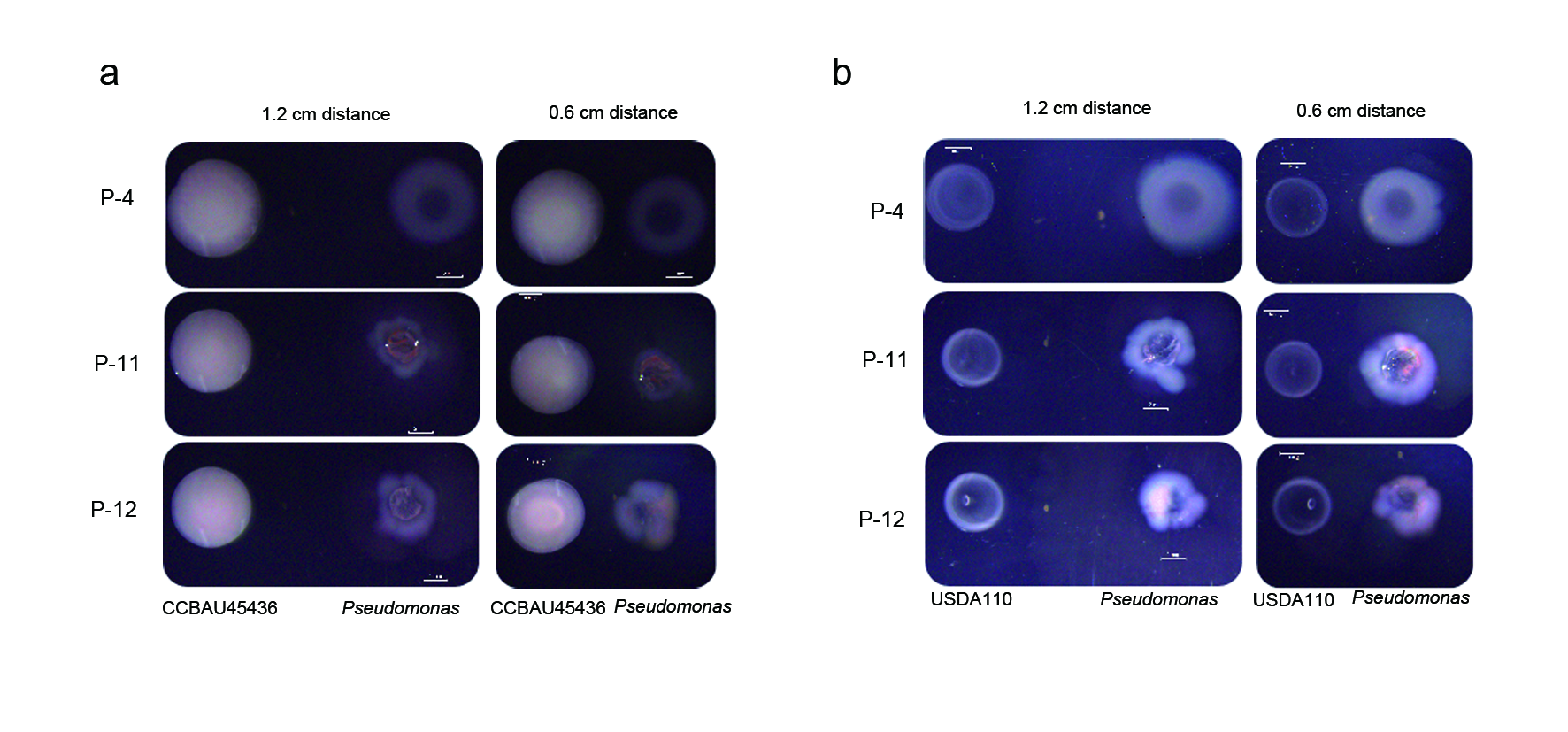

Supplement: Supplementary file 11 — Supplementary Fig S9 [file 41396_2020_648_MOESM11_ESM.tif]

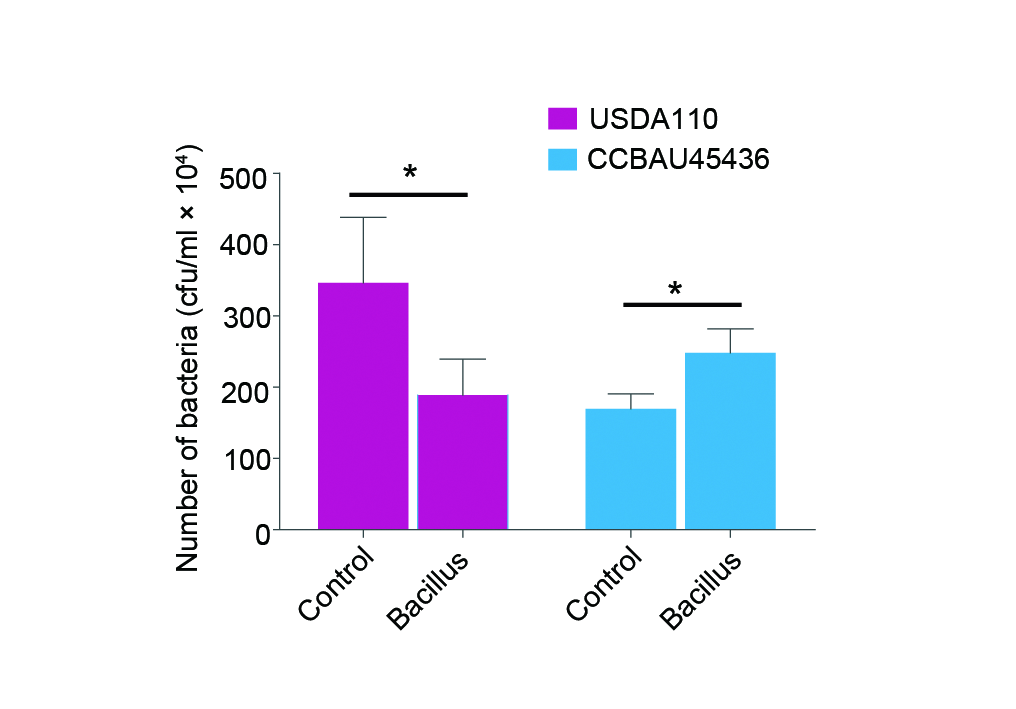

Supplement: Supplementary file 12 — Supplementary Fig S10 [file 41396_2020_648_MOESM12_ESM.tif]

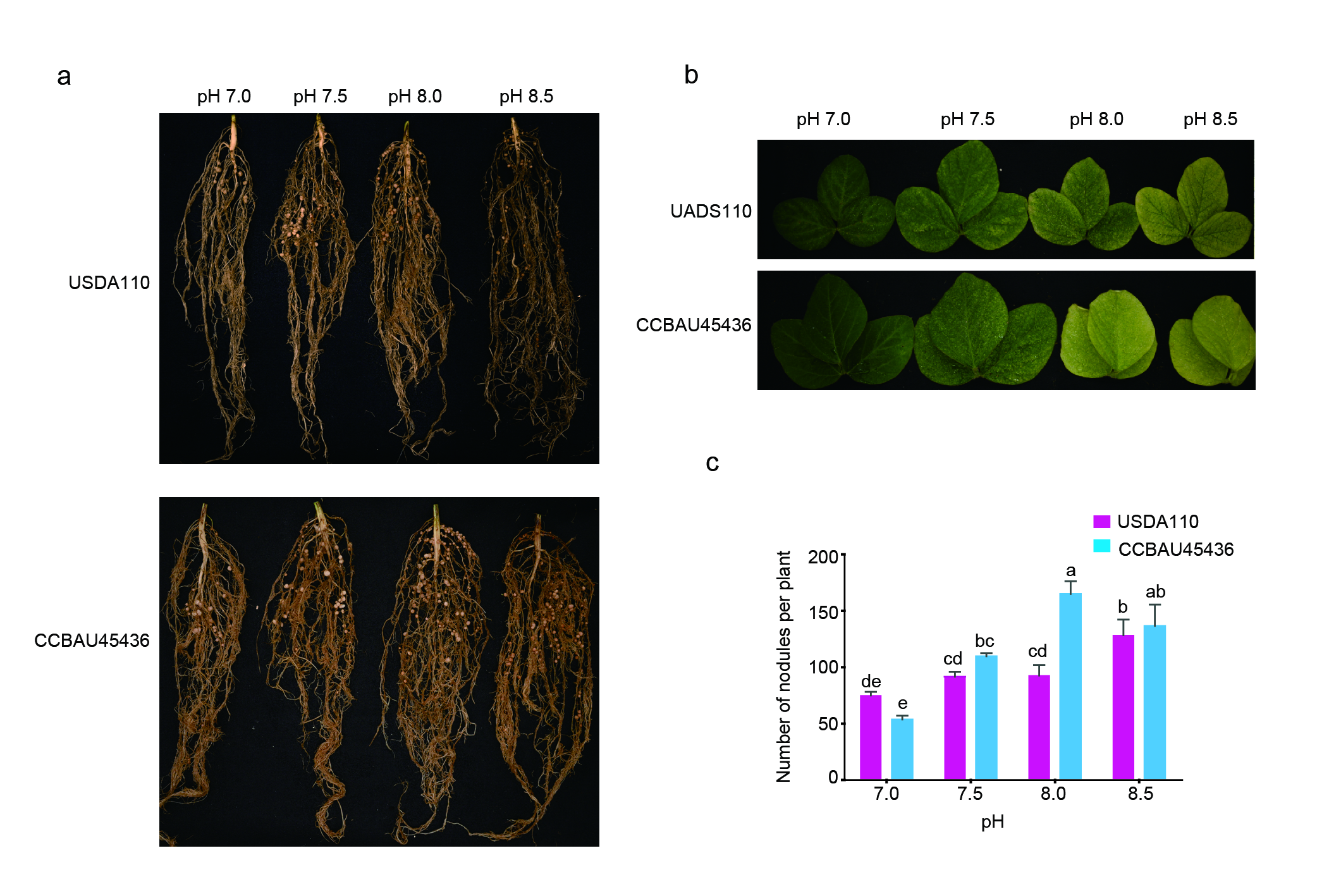

Supplement: Supplementary file 13 — Supplementary Fig S11 [file 41396_2020_648_MOESM13_ESM.tif]

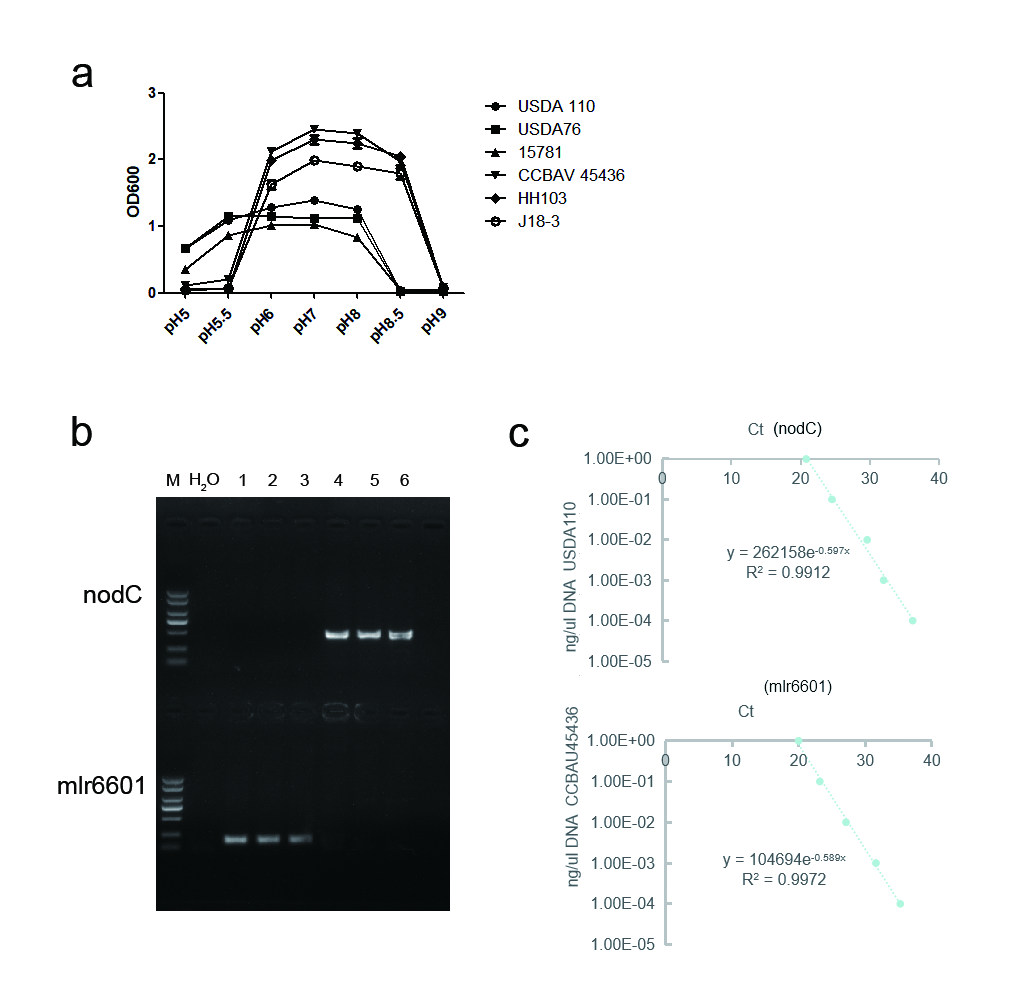

Supplement: Supplementary file 14 — Supplementary Fig S12 [file 41396_2020_648_MOESM14_ESM.tif]
