# Supplementary material for: Variation in rhizosphere microbial communities and its association with the symbiotic efficiency of rhizobia in soybean
Source: ISME J. 2020 Apr 27;14(8):1915–28. doi: 10.1038/s41396-020-0648-9 (PMC7367843; doi:10.1038/s41396-020-0648-9)
Supplement: Supplementary file 15 — Supplementary Fig S13 [file 41396_2020_648_MOESM15_ESM.doc]

Table S1 Soil chemical factors of sampling sites

| **Soil component** | **Wuhan** | **Siping** | **Luancheng** |
| --- | --- | --- | --- |
| pH | 5.63 | 7.2 | 8.23 |
| OM | 6.68 | 25.21 | 13.49 |
| N | 809.9 | 2487.9 | 1943.34 |
| P | 408.29 | 616.94 | 978.41 |
| K | 1.22 | 2.02 | 1.78 |
| Al | 5.8 | 5.54 | 7.62 |
| Fe | 4.32 | 2.86 | 2.66 |
| Ca | 0.12 | 0.97 | 2.65 |
| Na | 1.24 | 13.2 | 13.43 |
| NH4+ | 16.26 | 7.62 | 11.48 |
| NO3- | 2.7 | 19.59 | 14.49 |

pH, potential of hydrogen; OM, organic matter (mg/kg); N, nitrogen (mg/kg) P,

phosphorus(mg/kg); K, potassium (mg/kg); AI, aluminum (%); Fe, iron (%); Ca,

calcium (%); NH4+, Ammonium ion (mg/kg); NO3-, Nitrate ion (mg/kg).

Table S2 The number of replicates per sample in the greenhouse

| **Compartment** | **Untreated-soil** | | |  | **Treated-soil** | | | | | | |
| --- | --- | --- | --- | --- | --- | --- | --- | --- | --- | --- | --- |
|  | **Ac** | **Ne** | **Al** |  | **Ac8** | **Ne8** | **Ac/Al** | **Ne/Al** | **HAc** | **HNe** | **HAl** |
| Bulk soil | 4 | 4 | 4 |  |  |  |  |  |  |  |  |
| Root | 4 | 4 | 4 |  |  |  |  |  |  |  |  |
| Rhizosphere | 4 | 4 | 4 |  | 4 | 4 | 4 | 4 | 4 | 4 | 4 |
| Nodule | 4 | 4 | 4 |  | 4 | 4 | 4 | 4 | 4 | 4 | 4 |

Table S3 Specific qPCR primers designed for each resident bacterial species

| **Bacterial species** | **Forward primer (5' - 3')** | **Reverse primer (5' - 3')** | **Gene**  **[Reference]** |
| --- | --- | --- | --- |
| Bradyrhizioum | ATGCTCAAGGCGGGGTTTC | TCGCTATTGCTCAATGTACAAAG | *nodC* |
| Sinorhizobium | CTCGCGTCGGACTATGCA | CCACTTTCTCGGTCAGCTCTT | *mlr6601*[1] |

1. Gupta RS and Mok A. 2007. Phylogenomics and signature proteins for the alpha proteobacteria and its main groups. BMC Microbiol. 7. doi: 10.1186/1471-2180-7-106.

Table S4 The sequence number of pre treatment in untreated-soil samples

| **Compartment** | **Soil type** | **Seplicates** | **Seq_num** |
| --- | --- | --- | --- |
| Bulk soil | Ac | 1 | 38475 |
| Bulk soil | Ac | 2 | 34515 |
| Bulk soil | Ac | 3 | 30032 |
| Bulk soil | Ac | 4 | 42752 |
| Bulk soil | Ne | 1 | 43422 |
| Bulk soil | Ne | 2 | 38265 |
| Bulk soil | Ne | 3 | 39437 |
| Bulk soil | Ne | 4 | 43086 |
| Bulk soil | Al | 1 | 44608 |
| Bulk soil | Al | 2 | 42636 |
| Bulk soil | Al | 3 | 30252 |
| Bulk soil | Al | 4 | 38440 |
| Rhizosphere | Ac | 1 | 39547 |
| Rhizosphere | Ac | 2 | 33999 |
| Rhizosphere | Ac | 3 | 33628 |
| Rhizosphere | Ac | 4 | 38206 |
| Rhizosphere | Ne | 1 | 34809 |
| Rhizosphere | Ne | 2 | 34727 |
| Rhizosphere | Ne | 3 | 32557 |
| Rhizosphere | Ne | 4 | 43173 |
| Rhizosphere | Al | 1 | 37387 |
| Rhizosphere | Al | 2 | 31271 |
| Rhizosphere | Al | 3 | 44261 |
| Rhizosphere | Al | 4 | 31212 |
| Root | Ac | 1 | 36922 |
| Root | Ac | 2 | 34573 |
| Root | Ac | 3 | 33797 |
| Root | Ac | 4 | 43751 |
| Root | Ne | 1 | 36837 |
| Root | Ne | 2 | 41269 |
| Root | Ne | 3 | 40777 |
| Root | Ne | 4 | 43242 |
| Root | Al | 1 | 39268 |
| Root | Al | 2 | 42858 |
| Root | Al | 3 | 31047 |
| Root | Al | 4 | 39258 |
| Nodule | Ac | 1 | 40350 |
| Nodule | Ac | 2 | 33897 |
| Nodule | Ac | 3 | 37560 |
| Nodule | Ac | 4 | 32402 |
| Nodule | Ne | 1 | 39968 |
| Nodule | Ne | 2 | 37120 |
| Nodule | Ne | 3 | 31531 |
| Nodule | Ne | 4 | 30896 |
| Nodule | Al | 1 | 40841 |
| Nodule | Al | 2 | 34994 |
| Nodule | Al | 3 | 35795 |
| Nodule | Al | 4 | 38276 |

Table S5 General features of the high-throughput sequencing results in untreated-soil samples

| **Compartment** | **Number of sequence** | **Number of OTU97** | **Number of genera** | **Number of family** | **Number of order** | **Number of class** | **Number of phylum** | **Coverage** |
| --- | --- | --- | --- | --- | --- | --- | --- | --- |
|
| Bulk soil | 38827 | 2915 | 694 | 362 | 182 | 78 | 30 | 0.978 |
| Rhizosphere | 36231 | 2711 | 668 | 348 | 167 | 72 | 27 | 0.982 |
| Root | 38633 | 1377 | 456 | 237 | 109 | 46 | 23 | 0.991 |
| Nodule | 36136 | 1445 | 523 | 254 | 116 | 50 | 25 | 0.995 |

Table S6 PERMANOVA analysis of the rhizocompartment microbial community in different soil type composition based on Bray-Curtis

| **Factors** | **Df** | **SumsOfSqs** | **MeanSqs** | **F.Model** | **R2** | **Pr(>F)** | **Sig.** |
| --- | --- | --- | --- | --- | --- | --- | --- |
|  |  |  | **Whole Data** |  |  |  |  |
| Soil type | 2 | 3.2183 | 1.6092 | 5.9669 | 0.2656 | 0.001 | *** |
| Residuals | 33 | 8.8995 | 0.2697 | - | 0.7344 | - |  |
| Total | 35 | 12.1179 | - | - | 1 | - |  |
|  |  |  | **Rhizosphere** |  |  |  |  |
| Soil type | 2 | 1.906483701 | 0.95324185 | 26.87971418 | 0.856595252 | 0.001 | *** |
| Residuals | 9 | 0.319169192 | 0.035463244 | - | 0.143404748 | - |  |
| Total | 11 | 2.225652893 | - | - | 1 | - |  |
|  |  |  | **Root** |  |  |  |  |
| Soil type | 2 | 1.803774469 | 0.901887235 | 12.68292222 | 0.738112066 | 0.001 | *** |
| Residuals | 9 | 0.63999329 | 0.071110366 | - | 0.261887934 | - |  |
| Total | 11 | 2.443767759 | - | - | 1 | - |  |
|  |  |  | **Nodule** |  |  |  |  |
| Soil type | 2 | 2.368839471 | 1.184419736 | 23.3444655 | 0.838387991 | 0.007 | ** |
| Residuals | 9 | 0.456629757 | 0.05073664 | - | 0.161612009 |  |  |
| Total | 11 | 2.825469229 | - | - | 1 |  |  |

*, P < 0.05; **, P < 0.01; ***, P < 0.001.

Table S7 PERMANOVA analysis of the soybean microbial community in different soil type composition based on weighted UniFrac

| **Factors** | **Df** | **SumsOfSqs** | **MeanSqs** | **F.Model** | **R2** | **Pr(>F)** | **Sig.** |
| --- | --- | --- | --- | --- | --- | --- | --- |
|  |  |  | **Whole Data** |  |  |  |  |
| Soil type | 2 | 0.9983 | 0.4992 | 2.8925 | 0.1492 | 0.028 | * |
| Residuals | 33 | 5.6948 | 0.1726 | - | 0.8508 | - |  |
| Total | 35 | 6.6931 | - | - | 1 | - |  |
|  |  |  | **Rhizosphere** |  |  |  |  |
| Soil type | 2 | 1.906483701 | 0.95324185 | 26.87971418 | 0.856595252 | 0.001 | *** |
| Residuals | 9 | 0.319169192 | 0.035463244 | - | 0.143404748 | - |  |
| Total | 11 | 2.225652893 | - | - | 1 | - |  |
|  |  |  | **Root** |  |  |  |  |
| Soil type | 2 | 0.395997761 | 0.19799888 | 5.87886662 | 0.566426647 | 0.001 | *** |
| Residuals | 9 | 0.303117937 | 0.033679771 | - | 0.433573353 | - |  |
| Total | 11 | 0.699115698 | - | - | 1 | - |  |
|  |  |  | **Nodule** |  |  |  |  |
| Soil type | 2 | 0.654997652 | 0.327498826 | 8.794329264 | 0.661509813 | 0.005 | ** |
| Residuals | 9 | 0.335157958 | 0.037239773 | - | 0.338490187 | - |  |
| Total | 11 | 0.99015561 | - | - | 1 | - |  |

*, P < 0.05; **, P < 0.01; ***, P < 0.001.

**Table S8** Correlation coefficient of microorganisms (genus level) in bulk soil samples

**Node_Num = 29, Edge_Num = 202**

| **Node1_Name** | **Node2_Name** | **Coefficient** |
| --- | --- | --- |
| g__norank_c__Actinobacteria | g__Rubrobacter | 0.925413 |
| g__norank_c__Actinobacteria | g__norank_o__SC-I-84 | 0.671329 |
| g__norank_c__Actinobacteria | g__norank_f__Gemmatimonadaceae | 0.900177 |
| g__norank_c__Actinobacteria | g__unclassified_o__Solirubrobacterales | 0.893171 |
| g__norank_c__Actinobacteria | g__Gaiella | 0.942208 |
| g__norank_c__Actinobacteria | g__unclassified_f__Oxalobacteraceae | -0.858145 |
| g__norank_c__Actinobacteria | g__norank_o__Gaiellales | 0.944056 |
| g__norank_c__Actinobacteria | g__Nitrospira | 0.727273 |
| g__norank_c__Actinobacteria | g__Massilia | -0.93007 |
| g__norank_c__Actinobacteria | g__Solirubrobacter | 0.898251 |
| g__norank_c__Actinobacteria | g__Ramlibacter | -0.853147 |
| g__norank_c__Actinobacteria | g__Arthrobacter | -0.72028 |
| g__norank_c__Actinobacteria | g__Paucimonas | -0.902098 |
| g__Rubrobacter | g__norank_f__Gemmatimonadaceae | 0.869987 |
| g__Rubrobacter | g__unclassified_o__Solirubrobacterales | 0.969822 |
| g__Rubrobacter | g__Gaiella | 0.912773 |
| g__Rubrobacter | g__unclassified_f__Oxalobacteraceae | -0.841463 |
| g__Rubrobacter | g__norank_o__Gaiellales | 0.886261 |
| g__Rubrobacter | g__Nitrospira | 0.839991 |
| g__Rubrobacter | g__Massilia | -0.882702 |
| g__Rubrobacter | g__Solirubrobacter | 0.907235 |
| g__Rubrobacter | g__Bacillus | 0.605078 |
| g__Rubrobacter | g__Ramlibacter | -0.953888 |
| g__Rubrobacter | g__Arthrobacter | -0.669145 |
| g__Rubrobacter | g__Paucimonas | -0.854228 |
| g__norank_o__SC-I-84 | g__Gaiella | 0.65149 |
| g__norank_o__SC-I-84 | g__norank_f__Gemmatimonadaceae | 0.609458 |
| g__norank_o__SC-I-84 | g__norank_o__Gaiellales | 0.699301 |
| g__norank_f__Gemmatimonadaceae | g__norank_o__Acidimicrobiales | 0.680702 |
| g__norank_f__Gemmatimonadaceae | g__unclassified_o__Solirubrobacterales | 0.824561 |
| g__norank_f__Gemmatimonadaceae | g__Gaiella | 0.891228 |
| g__norank_f__Gemmatimonadaceae | g__unclassified_f__Oxalobacteraceae | -0.817544 |
| g__norank_f__Gemmatimonadaceae | g__norank_o__Gaiellales | 0.896674 |
| g__norank_f__Gemmatimonadaceae | g__Nitrospira | 0.711034 |
| g__norank_f__Gemmatimonadaceae | g__Massilia | -0.928198 |
| g__norank_f__Gemmatimonadaceae | g__Solirubrobacter | 0.748683 |
| g__norank_f__Gemmatimonadaceae | g__Ramlibacter | -0.875658 |
| g__norank_f__Gemmatimonadaceae | g__Arthrobacter | -0.830124 |
| g__norank_f__Gemmatimonadaceae | g__Paucimonas | -0.805605 |
| g__norank_o__Acidimicrobiales | g__Burkholderia-Paraburkholderia | -0.627417 |
| g__norank_o__Acidimicrobiales | g__Pseudarthrobacter | -0.711034 |
| g__norank_o__Acidimicrobiales | g__Nocardioides | 0.690019 |
| g__norank_o__Acidimicrobiales | g__norank_c__Acidobacteria | -0.701754 |
| g__norank_o__Acidimicrobiales | g__Bradyrhizobium | -0.626316 |
| g__norank_o__Acidimicrobiales | g__Lapillicoccus | -0.725045 |
| g__norank_o__Acidimicrobiales | g__Sphingomonas | -0.686516 |
| g__norank_o__Acidimicrobiales | g__Bacillus | 0.623469 |
| g__norank_o__Acidimicrobiales | g__norank_f__Nitrosomonadaceae | 0.763574 |
| g__norank_o__Acidimicrobiales | g__norank_f__Xanthobacteraceae | -0.619966 |
| g__norank_o__Acidimicrobiales | g__Arthrobacter | -0.690019 |
| g__norank_o__Acidimicrobiales | g__Microvirga | 0.638596 |
| g__norank_o__Acidimicrobiales | g__Blastococcus | 0.630474 |
| g__unclassified_o__Solirubrobacterales | g__Gaiella | 0.919298 |
| g__unclassified_o__Solirubrobacterales | g__unclassified_f__Oxalobacteraceae | -0.77193 |
| g__unclassified_o__Solirubrobacterales | g__norank_o__Gaiellales | 0.868653 |
| g__unclassified_o__Solirubrobacterales | g__Nitrospira | 0.788092 |
| g__unclassified_o__Solirubrobacterales | g__Massilia | -0.823119 |
| g__unclassified_o__Solirubrobacterales | g__Solirubrobacter | 0.896311 |
| g__unclassified_o__Solirubrobacterales | g__Ramlibacter | -0.945711 |
| g__unclassified_o__Solirubrobacterales | g__Arthrobacter | -0.654992 |
| g__unclassified_o__Solirubrobacterales | g__Paucimonas | -0.805605 |
| g__Burkholderia-Paraburkholderia | g__Pseudarthrobacter | 0.87369 |
| g__Burkholderia-Paraburkholderia | g__Nocardioides | -0.898251 |
| g__Burkholderia-Paraburkholderia | g__norank_c__Acidobacteria | 0.898068 |
| g__Burkholderia-Paraburkholderia | g__Bradyrhizobium | 0.950792 |
| g__Burkholderia-Paraburkholderia | g__Nitrospira | -0.652636 |
| g__Burkholderia-Paraburkholderia | g__norank_f__MSB-1E8 | -0.922267 |
| g__Burkholderia-Paraburkholderia | g__Lapillicoccus | 0.919304 |
| g__Burkholderia-Paraburkholderia | g__Sphingomonas | 0.831584 |
| g__Burkholderia-Paraburkholderia | g__Bacillus | -0.870181 |
| g__Burkholderia-Paraburkholderia | g__norank_f__Nitrosomonadaceae | -0.821058 |
| g__Burkholderia-Paraburkholderia | g__norank_f__Xanthobacteraceae | 0.884216 |
| g__Burkholderia-Paraburkholderia | g__Microvirga | -0.889281 |
| g__Burkholderia-Paraburkholderia | g__Paucimonas | 0.600004 |
| g__Burkholderia-Paraburkholderia | g__Gemmatimonas | 0.820739 |
| g__Burkholderia-Paraburkholderia | g__Blastococcus | -0.687724 |
| g__Gaiella | g__unclassified_f__Oxalobacteraceae | -0.85614 |
| g__Gaiella | g__norank_o__Gaiellales | 0.970229 |
| g__Gaiella | g__Nitrospira | 0.795098 |
| g__Gaiella | g__Massilia | -0.921192 |
| g__Gaiella | g__Solirubrobacter | 0.829527 |
| g__Gaiella | g__Ramlibacter | -0.896674 |
| g__Gaiella | g__Arthrobacter | -0.774082 |
| g__Gaiella | g__Paucimonas | -0.868653 |
| g__Pseudarthrobacter | g__Nocardioides | -0.888112 |
| g__Pseudarthrobacter | g__norank_c__Acidobacteria | 0.833626 |
| g__Pseudarthrobacter | g__Bradyrhizobium | 0.886166 |
| g__Pseudarthrobacter | g__norank_f__MSB-1E8 | -0.894388 |
| g__Pseudarthrobacter | g__Lapillicoccus | 0.902098 |
| g__Pseudarthrobacter | g__Sphingomonas | 0.895105 |
| g__Pseudarthrobacter | g__Bacillus | -0.706294 |
| g__Pseudarthrobacter | g__norank_f__Nitrosomonadaceae | -0.944056 |
| g__Pseudarthrobacter | g__norank_f__Xanthobacteraceae | 0.923077 |
| g__Pseudarthrobacter | g__Arthrobacter | 0.685315 |
| g__Pseudarthrobacter | g__Microvirga | -0.872155 |
| g__Pseudarthrobacter | g__Gemmatimonas | 0.711034 |
| g__Pseudarthrobacter | g__Blastococcus | -0.867133 |
| g__unclassified_f__Oxalobacteraceae | g__norank_o__Gaiellales | -0.858145 |
| g__unclassified_f__Oxalobacteraceae | g__Nitrospira | -0.728548 |
| g__unclassified_f__Oxalobacteraceae | g__Massilia | 0.928198 |
| g__unclassified_f__Oxalobacteraceae | g__Solirubrobacter | -0.650265 |
| g__unclassified_f__Oxalobacteraceae | g__Ramlibacter | 0.823119 |
| g__unclassified_f__Oxalobacteraceae | g__Arthrobacter | 0.619966 |
| g__unclassified_f__Oxalobacteraceae | g__Paucimonas | 0.795098 |
| g__Nocardioides | g__norank_c__Acidobacteria | -0.847637 |
| g__Nocardioides | g__Bradyrhizobium | -0.847637 |
| g__Nocardioides | g__Nitrospira | 0.713287 |
| g__Nocardioides | g__norank_f__MSB-1E8 | 0.894388 |
| g__Nocardioides | g__Lapillicoccus | -0.902098 |
| g__Nocardioides | g__Sphingomonas | -0.888112 |
| g__Nocardioides | g__Bacillus | 0.804196 |
| g__Nocardioides | g__norank_f__Nitrosomonadaceae | 0.797203 |
| g__Nocardioides | g__norank_f__Xanthobacteraceae | -0.846154 |
| g__Nocardioides | g__Microvirga | 0.973732 |
| g__Nocardioides | g__Gemmatimonas | -0.830124 |
| g__Nocardioides | g__Blastococcus | 0.692308 |
| g__norank_o__Gaiellales | g__Nitrospira | 0.762238 |
| g__norank_o__Gaiellales | g__Massilia | -0.895105 |
| g__norank_o__Gaiellales | g__Solirubrobacter | 0.821058 |
| g__norank_o__Gaiellales | g__Ramlibacter | -0.846154 |
| g__norank_o__Gaiellales | g__Arthrobacter | -0.783217 |
| g__norank_o__Gaiellales | g__Paucimonas | -0.867133 |
| g__norank_c__Acidobacteria | g__Bradyrhizobium | 0.929825 |
| g__norank_c__Acidobacteria | g__Nitrospira | -0.65149 |
| g__norank_c__Acidobacteria | g__norank_f__MSB-1E8 | -0.913593 |
| g__norank_c__Acidobacteria | g__Lapillicoccus | 0.872155 |
| g__norank_c__Acidobacteria | g__Sphingomonas | 0.91769 |
| g__norank_c__Acidobacteria | g__Bacillus | -0.9317 |
| g__norank_c__Acidobacteria | g__norank_f__Nitrosomonadaceae | -0.763574 |
| g__norank_c__Acidobacteria | g__norank_f__Xanthobacteraceae | 0.886166 |
| g__norank_c__Acidobacteria | g__Microvirga | -0.887719 |
| g__norank_c__Acidobacteria | g__Gemmatimonas | 0.92807 |
| g__norank_c__Acidobacteria | g__Blastococcus | -0.735553 |
| g__Bradyrhizobium | g__norank_f__MSB-1E8 | -0.941812 |
| g__Bradyrhizobium | g__Lapillicoccus | 0.914187 |
| g__Bradyrhizobium | g__Sphingomonas | 0.85114 |
| g__Bradyrhizobium | g__Bacillus | -0.86515 |
| g__Bradyrhizobium | g__norank_f__Nitrosomonadaceae | -0.823119 |
| g__Bradyrhizobium | g__norank_f__Xanthobacteraceae | 0.896674 |
| g__Bradyrhizobium | g__Microvirga | -0.870175 |
| g__Bradyrhizobium | g__Gemmatimonas | 0.849123 |
| g__Bradyrhizobium | g__Blastococcus | -0.767076 |
| g__Nitrospira | g__Massilia | -0.727273 |
| g__Nitrospira | g__Solirubrobacter | 0.733338 |
| g__Nitrospira | g__norank_f__MSB-1E8 | 0.626776 |
| g__Nitrospira | g__Lapillicoccus | -0.643357 |
| g__Nitrospira | g__Bacillus | 0.776224 |
| g__Nitrospira | g__Ramlibacter | -0.825175 |
| g__Nitrospira | g__Arthrobacter | -0.601399 |
| g__Nitrospira | g__Microvirga | 0.73205 |
| g__Nitrospira | g__Paucimonas | -0.79021 |
| g__Massilia | g__Solirubrobacter | -0.754391 |
| g__Massilia | g__Ramlibacter | 0.867133 |
| g__Massilia | g__Arthrobacter | 0.79021 |
| g__Massilia | g__Paucimonas | 0.86014 |
| g__Solirubrobacter | g__norank_f__MSB-1E8 | 0.611311 |
| g__Solirubrobacter | g__Ramlibacter | -0.831584 |
| g__Solirubrobacter | g__Arthrobacter | -0.6386 |
| g__Solirubrobacter | g__Paucimonas | -0.898251 |
| g__Solirubrobacter | g__Blastococcus | 0.6386 |
| g__norank_f__MSB-1E8 | g__Lapillicoccus | -0.873261 |
| g__norank_f__MSB-1E8 | g__Sphingomonas | -0.922558 |
| g__norank_f__MSB-1E8 | g__Bacillus | 0.838049 |
| g__norank_f__MSB-1E8 | g__norank_f__Nitrosomonadaceae | 0.788752 |
| g__norank_f__MSB-1E8 | g__norank_f__Xanthobacteraceae | -0.936643 |
| g__norank_f__MSB-1E8 | g__Microvirga | 0.94534 |
| g__norank_f__MSB-1E8 | g__Gemmatimonas | -0.835991 |
| g__norank_f__MSB-1E8 | g__Blastococcus | 0.823964 |
| g__Lapillicoccus | g__Sphingomonas | 0.818182 |
| g__Lapillicoccus | g__Bacillus | -0.825175 |
| g__Lapillicoccus | g__norank_f__Nitrosomonadaceae | -0.902098 |
| g__Lapillicoccus | g__norank_f__Xanthobacteraceae | 0.881119 |
| g__Lapillicoccus | g__Arthrobacter | 0.643357 |
| g__Lapillicoccus | g__Microvirga | -0.896674 |
| g__Lapillicoccus | g__Gemmatimonas | 0.746061 |
| g__Lapillicoccus | g__Blastococcus | -0.671329 |
| g__Sphingomonas | g__Bacillus | -0.776224 |
| g__Sphingomonas | g__norank_f__Nitrosomonadaceae | -0.818182 |
| g__Sphingomonas | g__norank_f__Xanthobacteraceae | 0.944056 |
| g__Sphingomonas | g__Microvirga | -0.914187 |
| g__Sphingomonas | g__Gemmatimonas | 0.837129 |
| g__Sphingomonas | g__Blastococcus | -0.853147 |
| g__Bacillus | g__norank_f__Nitrosomonadaceae | 0.643357 |
| g__Bacillus | g__Ramlibacter | -0.657343 |
| g__Bacillus | g__norank_f__Xanthobacteraceae | -0.762238 |
| g__Bacillus | g__Microvirga | 0.837129 |
| g__Bacillus | g__Paucimonas | -0.601399 |
| g__Bacillus | g__Gemmatimonas | -0.882663 |
| g__norank_f__Nitrosomonadaceae | g__norank_f__Xanthobacteraceae | -0.909091 |
| g__norank_f__Nitrosomonadaceae | g__Arthrobacter | -0.797203 |
| g__norank_f__Nitrosomonadaceae | g__Microvirga | 0.763574 |
| g__norank_f__Nitrosomonadaceae | g__Blastococcus | 0.797203 |
| g__Ramlibacter | g__Arthrobacter | 0.678322 |
| g__Ramlibacter | g__Paucimonas | 0.839161 |
| g__norank_f__Xanthobacteraceae | g__Arthrobacter | 0.629371 |
| g__norank_f__Xanthobacteraceae | g__Microvirga | -0.872155 |
| g__norank_f__Xanthobacteraceae | g__Gemmatimonas | 0.728548 |
| g__norank_f__Xanthobacteraceae | g__Blastococcus | -0.839161 |
| g__Arthrobacter | g__Paucimonas | 0.727273 |
| g__Arthrobacter | g__Blastococcus | -0.664336 |
| g__Microvirga | g__Gemmatimonas | -0.852632 |
| g__Microvirga | g__Blastococcus | 0.739055 |

**Table S9** Correlation coefficient of microorganisms (genus level) in rhizosphere samples

Node_Num = 28, Edge_Num = 184

| **Node1_Name** | **Node2_Name** | **Coefficient** |
| --- | --- | --- |
| g__norank_c__Actinobacteria | g__Rubrobacter | 0.98594 |
| g__norank_c__Actinobacteria | g__norank_o__SC-I-84 | 0.888112 |
| g__norank_c__Actinobacteria | g__norank_f__Gemmatimonadaceae | 0.923077 |
| g__norank_c__Actinobacteria | g__Methylobacterium | -0.854142 |
| g__norank_c__Actinobacteria | g__Gaiella | 0.979021 |
| g__norank_c__Actinobacteria | g__norank_o__Gaiellales | 0.965035 |
| g__norank_c__Actinobacteria | g__Ralstonia | -0.832168 |
| g__norank_c__Actinobacteria | g__Massilia | -0.615385 |
| g__norank_c__Actinobacteria | g__Bacillus | 0.706294 |
| g__norank_c__Actinobacteria | g__norank_f__Nitrosomonadaceae | 0.690019 |
| g__norank_c__Actinobacteria | g__Ramlibacter | -0.895105 |
| g__norank_c__Actinobacteria | g__Streptomyces | 0.615385 |
| g__norank_c__Actinobacteria | g__Blastococcus | 0.629371 |
| g__Rubrobacter | g__norank_o__SC-I-84 | 0.929601 |
| g__Rubrobacter | g__norank_f__Gemmatimonadaceae | 0.964813 |
| g__Rubrobacter | g__Methylobacterium | -0.853099 |
| g__Rubrobacter | g__Variovorax | 0.60671 |
| g__Rubrobacter | g__Gaiella | 0.964813 |
| g__Rubrobacter | g__norank_o__Gaiellales | 0.964813 |
| g__Rubrobacter | g__Ralstonia | -0.873261 |
| g__Rubrobacter | g__Bacillus | 0.711285 |
| g__Rubrobacter | g__norank_f__Nitrosomonadaceae | 0.673731 |
| g__Rubrobacter | g__Ramlibacter | -0.901431 |
| g__Rubrobacter | g__Streptomyces | 0.626776 |
| g__Rubrobacter | g__Blastococcus | 0.640861 |
| g__Enterobacter | g__Mesorhizobium | -0.784496 |
| g__Enterobacter | g__Burkholderia-Paraburkholderia | -0.817183 |
| g__Enterobacter | g__Pseudarthrobacter | -0.761369 |
| g__Enterobacter | g__Nocardioides | 0.739616 |
| g__Enterobacter | g__Bradyrhizobium | -0.862885 |
| g__Enterobacter | g__Massilia | -0.732365 |
| g__Enterobacter | g__Sphingomonas | -0.732365 |
| g__Enterobacter | g__norank_f__Nitrosomonadaceae | 0.606531 |
| g__Enterobacter | g__Phenylobacterium | -0.746867 |
| g__Enterobacter | g__Azotobacter | 0.744544 |
| g__Enterobacter | g__Roseateles | 0.739616 |
| g__Enterobacter | g__Sinorhizobium | 0.726385 |
| g__Mesorhizobium | g__Burkholderia-Paraburkholderia | 0.750877 |
| g__Mesorhizobium | g__Bradyrhizobium | 0.795098 |
| g__Mesorhizobium | g__Massilia | 0.763574 |
| g__Mesorhizobium | g__Rhizobium | 0.760071 |
| g__Mesorhizobium | g__Sphingomonas | 0.795098 |
| g__Mesorhizobium | g__norank_f__Nitrosomonadaceae | -0.757895 |
| g__Mesorhizobium | g__Phenylobacterium | 0.602453 |
| g__Mesorhizobium | g__Blastococcus | -0.697024 |
| g__norank_o__SC-I-84 | g__norank_f__Gemmatimonadaceae | 0.93007 |
| g__norank_o__SC-I-84 | g__Methylobacterium | -0.794387 |
| g__norank_o__SC-I-84 | g__Variovorax | 0.714537 |
| g__norank_o__SC-I-84 | g__Gaiella | 0.895105 |
| g__norank_o__SC-I-84 | g__unclassified_f__Oxalobacteraceae | 0.608392 |
| g__norank_o__SC-I-84 | g__norank_o__Gaiellales | 0.909091 |
| g__norank_o__SC-I-84 | g__Ralstonia | -0.888112 |
| g__norank_o__SC-I-84 | g__Bacillus | 0.72028 |
| g__norank_o__SC-I-84 | g__norank_f__Nitrosomonadaceae | 0.676008 |
| g__norank_o__SC-I-84 | g__Ramlibacter | -0.881119 |
| g__norank_o__SC-I-84 | g__Blastococcus | 0.699301 |
| g__norank_f__Gemmatimonadaceae | g__Methylobacterium | -0.882262 |
| g__norank_f__Gemmatimonadaceae | g__Variovorax | 0.763574 |
| g__norank_f__Gemmatimonadaceae | g__Gaiella | 0.93007 |
| g__norank_f__Gemmatimonadaceae | g__norank_o__Gaiellales | 0.951049 |
| g__norank_f__Gemmatimonadaceae | g__Ralstonia | -0.937063 |
| g__norank_f__Gemmatimonadaceae | g__Bacillus | 0.636364 |
| g__norank_f__Gemmatimonadaceae | g__norank_f__Nitrosomonadaceae | 0.661998 |
| g__norank_f__Gemmatimonadaceae | g__Ramlibacter | -0.867133 |
| g__norank_f__Gemmatimonadaceae | g__Streptomyces | 0.601399 |
| g__norank_f__Gemmatimonadaceae | g__Blastococcus | 0.657343 |
| g__Methylobacterium | g__Burkholderia-Paraburkholderia | 0.676061 |
| g__Methylobacterium | g__Gaiella | -0.903352 |
| g__Methylobacterium | g__unclassified_f__Oxalobacteraceae | -0.622153 |
| g__Methylobacterium | g__norank_o__Gaiellales | -0.854142 |
| g__Methylobacterium | g__Ralstonia | 0.854142 |
| g__Methylobacterium | g__Massilia | 0.667848 |
| g__Methylobacterium | g__Bacillus | -0.618638 |
| g__Methylobacterium | g__norank_f__Nitrosomonadaceae | -0.697187 |
| g__Methylobacterium | g__Phenylobacterium | 0.615123 |
| g__Methylobacterium | g__Ramlibacter | 0.811962 |
| g__Methylobacterium | g__Blastococcus | -0.625668 |
| g__Variovorax | g__norank_o__Gaiellales | 0.609458 |
| g__Variovorax | g__Ralstonia | -0.791595 |
| g__Burkholderia-Paraburkholderia | g__Pseudarthrobacter | 0.868653 |
| g__Burkholderia-Paraburkholderia | g__unclassified_f__Oxalobacteraceae | -0.795098 |
| g__Burkholderia-Paraburkholderia | g__Nocardioides | -0.86515 |
| g__Burkholderia-Paraburkholderia | g__Bradyrhizobium | 0.91769 |
| g__Burkholderia-Paraburkholderia | g__Massilia | 0.819616 |
| g__Burkholderia-Paraburkholderia | g__Sphingomonas | 0.819616 |
| g__Burkholderia-Paraburkholderia | g__norank_f__Nitrosomonadaceae | -0.822807 |
| g__Burkholderia-Paraburkholderia | g__Phenylobacterium | 0.914187 |
| g__Burkholderia-Paraburkholderia | g__Roseateles | -0.900177 |
| g__Burkholderia-Paraburkholderia | g__Sinorhizobium | -0.817544 |
| g__Burkholderia-Paraburkholderia | g__Blastococcus | -0.781087 |
| g__Gaiella | g__norank_o__Gaiellales | 0.972028 |
| g__Gaiella | g__Ralstonia | -0.867133 |
| g__Gaiella | g__Bacillus | 0.671329 |
| g__Gaiella | g__norank_f__Nitrosomonadaceae | 0.693521 |
| g__Gaiella | g__Phenylobacterium | -0.608392 |
| g__Gaiella | g__Ramlibacter | -0.874126 |
| g__Gaiella | g__Blastococcus | 0.643357 |
| g__Pseudarthrobacter | g__unclassified_f__Oxalobacteraceae | -0.811189 |
| g__Pseudarthrobacter | g__Nocardioides | -0.888112 |
| g__Pseudarthrobacter | g__Bradyrhizobium | 0.853147 |
| g__Pseudarthrobacter | g__Massilia | 0.699301 |
| g__Pseudarthrobacter | g__Sphingomonas | 0.685315 |
| g__Pseudarthrobacter | g__norank_f__Nitrosomonadaceae | -0.711034 |
| g__Pseudarthrobacter | g__Phenylobacterium | 0.776224 |
| g__Pseudarthrobacter | g__Azotobacter | -0.643244 |
| g__Pseudarthrobacter | g__Roseateles | -0.881119 |
| g__Pseudarthrobacter | g__Streptomyces | -0.699301 |
| g__Pseudarthrobacter | g__Sinorhizobium | -0.858145 |
| g__Pseudarthrobacter | g__Blastococcus | -0.734266 |
| g__unclassified_f__Oxalobacteraceae | g__Nocardioides | 0.881119 |
| g__unclassified_f__Oxalobacteraceae | g__Ralstonia | -0.664336 |
| g__unclassified_f__Oxalobacteraceae | g__Bradyrhizobium | -0.776224 |
| g__unclassified_f__Oxalobacteraceae | g__Sphingomonas | -0.783217 |
| g__unclassified_f__Oxalobacteraceae | g__Bacillus | 0.664336 |
| g__unclassified_f__Oxalobacteraceae | g__norank_f__Nitrosomonadaceae | 0.879161 |
| g__unclassified_f__Oxalobacteraceae | g__Phenylobacterium | -0.72028 |
| g__unclassified_f__Oxalobacteraceae | g__Roseateles | 0.804196 |
| g__unclassified_f__Oxalobacteraceae | g__Sinorhizobium | 0.746061 |
| g__unclassified_f__Oxalobacteraceae | g__Blastococcus | 0.951049 |
| g__Nocardioides | g__Bradyrhizobium | -0.895105 |
| g__Nocardioides | g__Massilia | -0.636364 |
| g__Nocardioides | g__Sphingomonas | -0.776224 |
| g__Nocardioides | g__Bacillus | 0.692308 |
| g__Nocardioides | g__norank_f__Nitrosomonadaceae | 0.861648 |
| g__Nocardioides | g__Phenylobacterium | -0.881119 |
| g__Nocardioides | g__Azotobacter | 0.762917 |
| g__Nocardioides | g__Roseateles | 0.867133 |
| g__Nocardioides | g__Streptomyces | 0.678322 |
| g__Nocardioides | g__Sinorhizobium | 0.816113 |
| g__Nocardioides | g__Blastococcus | 0.874126 |
| g__norank_o__Gaiellales | g__Ralstonia | -0.909091 |
| g__norank_o__Gaiellales | g__Bacillus | 0.643357 |
| g__norank_o__Gaiellales | g__norank_f__Nitrosomonadaceae | 0.630474 |
| g__norank_o__Gaiellales | g__Ramlibacter | -0.916084 |
| g__norank_o__Gaiellales | g__Streptomyces | 0.636364 |
| g__norank_o__Gaiellales | g__Blastococcus | 0.601399 |
| g__Ralstonia | g__Bacillus | -0.692308 |
| g__Ralstonia | g__Ramlibacter | 0.825175 |
| g__Ralstonia | g__Streptomyces | -0.699301 |
| g__Ralstonia | g__Blastococcus | -0.643357 |
| g__Bradyrhizobium | g__Massilia | 0.804196 |
| g__Bradyrhizobium | g__Sphingomonas | 0.867133 |
| g__Bradyrhizobium | g__norank_f__Nitrosomonadaceae | -0.86515 |
| g__Bradyrhizobium | g__Phenylobacterium | 0.825175 |
| g__Bradyrhizobium | g__Azotobacter | -0.673162 |
| g__Bradyrhizobium | g__Roseateles | -0.818182 |
| g__Bradyrhizobium | g__Sinorhizobium | -0.704029 |
| g__Bradyrhizobium | g__Blastococcus | -0.783217 |
| g__Massilia | g__Sphingomonas | 0.629371 |
| g__Massilia | g__norank_f__Nitrosomonadaceae | -0.693521 |
| g__Massilia | g__Phenylobacterium | 0.783217 |
| g__Massilia | g__Ramlibacter | 0.629371 |
| g__Massilia | g__Roseateles | -0.713287 |
| g__Rhizobium | g__Sphingomonas | 0.685315 |
| g__Rhizobium | g__norank_f__Nitrosomonadaceae | -0.658495 |
| g__Sphingomonas | g__norank_f__Nitrosomonadaceae | -0.837129 |
| g__Sphingomonas | g__Phenylobacterium | 0.706294 |
| g__Sphingomonas | g__Azotobacter | -0.684381 |
| g__Sphingomonas | g__Roseateles | -0.783217 |
| g__Sphingomonas | g__Sinorhizobium | -0.669003 |
| g__Sphingomonas | g__Blastococcus | -0.79021 |
| g__Bacillus | g__norank_f__Nitrosomonadaceae | 0.602453 |
| g__Bacillus | g__Phenylobacterium | -0.741259 |
| g__Bacillus | g__Ramlibacter | -0.699301 |
| g__Bacillus | g__Roseateles | 0.762238 |
| g__Bacillus | g__Streptomyces | 0.776224 |
| g__Bacillus | g__Sinorhizobium | 0.714537 |
| g__Bacillus | g__Blastococcus | 0.636364 |
| g__norank_f__Nitrosomonadaceae | g__Phenylobacterium | -0.767076 |
| g__norank_f__Nitrosomonadaceae | g__Roseateles | 0.700526 |
| g__norank_f__Nitrosomonadaceae | g__Blastococcus | 0.942208 |
| g__Phenylobacterium | g__Azotobacter | -0.71056 |
| g__Phenylobacterium | g__Roseateles | -0.874126 |
| g__Phenylobacterium | g__Streptomyces | -0.643357 |
| g__Phenylobacterium | g__Sinorhizobium | -0.830124 |
| g__Phenylobacterium | g__Blastococcus | -0.72028 |
| g__Ramlibacter | g__Streptomyces | -0.664336 |
| g__Azotobacter | g__Roseateles | 0.695601 |
| g__Azotobacter | g__Sinorhizobium | 0.756761 |
| g__Roseateles | g__Streptomyces | 0.692308 |
| g__Roseateles | g__Sinorhizobium | 0.959721 |
| g__Roseateles | g__Blastococcus | 0.734266 |
| g__Streptomyces | g__Sinorhizobium | 0.746061 |
| g__Sinorhizobium | g__Blastococcus | 0.647987 |

**Table S10** Correlation coefficient of microorganisms (genus level) in root samples

Node_Num = 28, Edge_Num = 90

| **Node1_Name** | **Node2_Name** | **Coefficient** |
| --- | --- | --- |
| g__unclassified_f__Micromonosporaceae | g__Ralstonia | -0.811189 |
| g__unclassified_f__Micromonosporaceae | g__Verrucosispora | 0.991245 |
| g__unclassified_f__Micromonosporaceae | g__Methylobacterium | -0.661998 |
| g__unclassified_f__Micromonosporaceae | g__Variovorax | 0.629371 |
| g__Verrucosispora | g__Ralstonia | -0.756569 |
| g__Methylobacterium | g__Pseudomonas | -0.647987 |
| g__Methylobacterium | g__Variovorax | -0.875658 |
| g__Methylobacterium | g__Burkholderia-Paraburkholderia | 0.677193 |
| g__Methylobacterium | g__unclassified_o__Rhizobiales | 0.811156 |
| g__Methylobacterium | g__Ralstonia | 0.679511 |
| g__Methylobacterium | g__Actinoplanes | -0.619966 |
| g__Methylobacterium | g__Phenylobacterium | 0.704029 |
| g__Methylobacterium | g__Roseateles | -0.739055 |
| g__Pseudomonas | g__unclassified_f__Oxalobacteraceae | 0.727273 |
| g__Pseudomonas | g__Bradyrhizobium | -0.713287 |
| g__Pseudomonas | g__unclassified_f__Comamonadaceae | 0.636364 |
| g__Pseudomonas | g__Roseateles | 0.608392 |
| g__Aquincola | g__Acidovorax | -0.859176 |
| g__Aquincola | g__Burkholderia-Paraburkholderia | 0.93123 |
| g__Aquincola | g__Caulobacter | 0.744278 |
| g__Aquincola | g__Nocardioides | -0.694895 |
| g__Aquincola | g__unclassified_o__Rhizobiales | 0.752732 |
| g__Aquincola | g__Massilia | 0.795794 |
| g__Aquincola | g__Sphingomonas | 0.767625 |
| g__Aquincola | g__Actinoplanes | -0.816922 |
| g__Aquincola | g__Phenylobacterium | 0.767625 |
| g__Aquincola | g__Roseateles | -0.739455 |
| g__Aquincola | g__Microbacterium | -0.774667 |
| g__Acidovorax | g__Variovorax | 0.608392 |
| g__Acidovorax | g__Burkholderia-Paraburkholderia | -0.9317 |
| g__Acidovorax | g__unclassified_o__Rhizobiales | -0.822194 |
| g__Acidovorax | g__Sphingomonas | -0.65035 |
| g__Acidovorax | g__Actinoplanes | 0.86014 |
| g__Acidovorax | g__Phenylobacterium | -0.832168 |
| g__Acidovorax | g__Roseateles | 0.902098 |
| g__Acidovorax | g__Microbacterium | 0.804196 |
| g__Variovorax | g__unclassified_o__Rhizobiales | -0.733212 |
| g__Variovorax | g__Ralstonia | -0.636364 |
| g__Variovorax | g__Phenylobacterium | -0.629371 |
| g__Variovorax | g__Roseateles | 0.664336 |
| g__Burkholderia-Paraburkholderia | g__Caulobacter | 0.624561 |
| g__Burkholderia-Paraburkholderia | g__unclassified_o__Rhizobiales | 0.855725 |
| g__Burkholderia-Paraburkholderia | g__Bradyrhizobium | 0.616463 |
| g__Burkholderia-Paraburkholderia | g__Massilia | 0.623469 |
| g__Burkholderia-Paraburkholderia | g__Sphingomonas | 0.753066 |
| g__Burkholderia-Paraburkholderia | g__Actinoplanes | -0.882663 |
| g__Burkholderia-Paraburkholderia | g__Phenylobacterium | 0.879161 |
| g__Burkholderia-Paraburkholderia | g__Roseateles | -0.889669 |
| g__Burkholderia-Paraburkholderia | g__Microbacterium | -0.781087 |
| g__Caulobacter | g__Enterobacter | -0.635088 |
| g__Caulobacter | g__Nocardioides | -0.685965 |
| g__Caulobacter | g__Sphingomonas | 0.896674 |
| g__Caulobacter | g__Microbacterium | -0.676008 |
| g__Caulobacter | g__Rhizobacter | 0.697024 |
| g__Caulobacter | g__Novosphingobium | 0.73205 |
| g__Enterobacter | g__Rhizobacter | -0.73205 |
| g__Enterobacter | g__Nocardioides | 0.645614 |
| g__Enterobacter | g__unclassified_f__Enterobacteriaceae | 0.802103 |
| g__Enterobacter | g__Bradyrhizobium | -0.612961 |
| g__unclassified_f__Oxalobacteraceae | g__Pelomonas | 0.804196 |
| g__Nocardioides | g__Massilia | -0.65149 |
| g__Nocardioides | g__Actinoplanes | 0.658495 |
| g__Nocardioides | g__Microbacterium | 0.686516 |
| g__Nocardioides | g__unclassified_f__Enterobacteriaceae | 0.623469 |
| g__Pelomonas | g__Massilia | 0.699301 |
| g__unclassified_o__Rhizobiales | g__Ralstonia | 0.665586 |
| g__unclassified_o__Rhizobiales | g__Actinoplanes | -0.658467 |
| g__unclassified_o__Rhizobiales | g__Phenylobacterium | 0.936091 |
| g__unclassified_o__Rhizobiales | g__Roseateles | -0.779483 |
| g__Ralstonia | g__Phenylobacterium | 0.685315 |
| g__Bradyrhizobium | g__Mesorhizobium | 0.657343 |
| g__Bradyrhizobium | g__Actinoplanes | -0.629371 |
| g__Bradyrhizobium | g__Roseateles | -0.741259 |
| g__Bradyrhizobium | g__unclassified_f__Enterobacteriaceae | -0.636364 |
| g__Mesorhizobium | g__Rhizobium | 0.741259 |
| g__Massilia | g__Actinoplanes | -0.657343 |
| g__Massilia | g__Microbacterium | -0.622378 |
| g__Sphingomonas | g__Phenylobacterium | 0.692308 |
| g__Sphingomonas | g__Roseateles | -0.615385 |
| g__Sphingomonas | g__Microbacterium | -0.776224 |
| g__Sphingomonas | g__Rhizobacter | 0.657343 |
| g__Sphingomonas | g__Novosphingobium | 0.72028 |
| g__Actinoplanes | g__Phenylobacterium | -0.657343 |
| g__Actinoplanes | g__Roseateles | 0.902098 |
| g__Actinoplanes | g__Microbacterium | 0.685315 |
| g__Phenylobacterium | g__Roseateles | -0.79021 |
| g__Phenylobacterium | g__Microbacterium | -0.608392 |
| g__Phenylobacterium | g__Novosphingobium | 0.706294 |
| g__Roseateles | g__Microbacterium | 0.741259 |
| g__Rhizobacter | g__unclassified_f__Enterobacteriaceae | -0.601399 |

**Table S11** Correlation coefficient of microorganisms (genus level) in nodule samples

Node_Num = 30, Edge_Num = 185

| **Node1_Name** | **Node2_Name** | **Coefficient** |
| --- | --- | --- |
| g__Prevotella_1 | g__Subdoligranulum | 0.738549 |
| g__Prevotella_1 | g__Bacteroides | 1 |
| g__Prevotella_1 | g__norank_f__Bacteroidales_S24-7_group | 0.738549 |
| g__Prevotella_1 | g__Faecalibacterium | 0.738549 |
| g__Prevotella_1 | g__Lactobacillus | 0.738549 |
| g__Bacteroides | g__Lactobacillus | 0.738549 |
| g__Bacteroides | g__Faecalibacterium | 0.738549 |
| g__Bacteroides | g__norank_f__Bacteroidales_S24-7_group | 0.738549 |
| g__Bacteroides | g__Subdoligranulum | 0.738549 |
| g__Pseudomonas | g__Noviherbaspirillum | 0.748422 |
| g__Pseudomonas | g__Variovorax | 0.827355 |
| g__Pseudomonas | g__Nocardioides | 0.884146 |
| g__Pseudomonas | g__Ralstonia | 0.729177 |
| g__Pseudomonas | g__Prevotella_9 | 0.628096 |
| g__Pseudomonas | g__Rhizobium | 0.915285 |
| g__Pseudomonas | g__Sphingomonas | 0.812006 |
| g__Pseudomonas | g__Bacillus | 0.66444 |
| g__Pseudomonas | g__Methylotenera | 0.790962 |
| g__Pseudomonas | g__Phenylobacterium | 0.845162 |
| g__Pseudomonas | g__Roseateles | 0.849624 |
| g__Pseudomonas | g__Arthrobacter | 0.627983 |
| g__Pseudomonas | g__Streptomyces | 0.815663 |
| g__Pseudomonas | g__Rhodococcus | 0.834586 |
| g__Pseudomonas | g__Escherichia-Shigella | 0.638477 |
| g__Pseudomonas | g__Novosphingobium | 0.919341 |
| g__Pseudomonas | g__unclassified_f__Alcaligenaceae | 0.806028 |
| g__Noviherbaspirillum | g__Variovorax | 0.828983 |
| g__Noviherbaspirillum | g__Burkholderia-Paraburkholderia | 0.653357 |
| g__Noviherbaspirillum | g__Nocardioides | 0.662 |
| g__Noviherbaspirillum | g__Ralstonia | 0.891996 |
| g__Noviherbaspirillum | g__Rhizobium | 0.879402 |
| g__Noviherbaspirillum | g__Sphingomonas | 0.754583 |
| g__Noviherbaspirillum | g__Methylotenera | 0.705154 |
| g__Noviherbaspirillum | g__Phenylobacterium | 0.822298 |
| g__Noviherbaspirillum | g__Roseateles | 0.814345 |
| g__Noviherbaspirillum | g__Arthrobacter | 0.759802 |
| g__Noviherbaspirillum | g__Streptomyces | 0.771561 |
| g__Noviherbaspirillum | g__Rhodococcus | 0.814345 |
| g__Noviherbaspirillum | g__Novosphingobium | 0.792782 |
| g__Noviherbaspirillum | g__unclassified_f__Alcaligenaceae | 0.61191 |
| g__Variovorax | g__Nocardioides | 0.759431 |
| g__Variovorax | g__Ralstonia | 0.760224 |
| g__Variovorax | g__Rhizobium | 0.908837 |
| g__Variovorax | g__Sphingomonas | 0.71277 |
| g__Variovorax | g__Bacillus | 0.625339 |
| g__Variovorax | g__Methylotenera | 0.695631 |
| g__Variovorax | g__Phenylobacterium | 0.758557 |
| g__Variovorax | g__Roseateles | 0.798197 |
| g__Variovorax | g__Arthrobacter | 0.769652 |
| g__Variovorax | g__Streptomyces | 0.819154 |
| g__Variovorax | g__Rhodococcus | 0.805486 |
| g__Variovorax | g__Novosphingobium | 0.830693 |
| g__Variovorax | g__unclassified_f__Alcaligenaceae | 0.618947 |
| g__Burkholderia-Paraburkholderia | g__Bifidobacterium | 0.664534 |
| g__Burkholderia-Paraburkholderia | g__Ralstonia | 0.67513 |
| g__Burkholderia-Paraburkholderia | g__Prevotella_9 | 0.6711 |
| g__Burkholderia-Paraburkholderia | g__Roseateles | 0.607474 |
| g__Burkholderia-Paraburkholderia | g__Streptomyces | 0.636087 |
| g__Bifidobacterium | g__Nocardioides | 0.664078 |
| g__Bifidobacterium | g__Subdoligranulum | 0.738549 |
| g__Bifidobacterium | g__Ralstonia | 0.657376 |
| g__Bifidobacterium | g__Rhizobium | 0.601929 |
| g__Bifidobacterium | g__Sphingomonas | 0.656205 |
| g__Bifidobacterium | g__Bacillus | 0.697097 |
| g__Bifidobacterium | g__Lactobacillus | 0.738549 |
| g__Bifidobacterium | g__Phenylobacterium | 0.601929 |
| g__Bifidobacterium | g__Roseateles | 0.663305 |
| g__Bifidobacterium | g__norank_f__Bacteroidales_S24-7_group | 0.738549 |
| g__Bifidobacterium | g__Arthrobacter | 0.708572 |
| g__Bifidobacterium | g__Rhodococcus | 0.674453 |
| g__Bifidobacterium | g__Faecalibacterium | 0.738549 |
| g__Bifidobacterium | g__Escherichia-Shigella | 0.773957 |
| g__Nocardioides | g__Ralstonia | 0.820183 |
| g__Nocardioides | g__Prevotella_9 | 0.727816 |
| g__Nocardioides | g__Rhizobium | 0.875595 |
| g__Nocardioides | g__Sphingomonas | 0.886635 |
| g__Nocardioides | g__Bacillus | 0.799601 |
| g__Nocardioides | g__Methylotenera | 0.751775 |
| g__Nocardioides | g__Phenylobacterium | 0.883209 |
| g__Nocardioides | g__Roseateles | 0.926802 |
| g__Nocardioides | g__Arthrobacter | 0.68647 |
| g__Nocardioides | g__Streptomyces | 0.801744 |
| g__Nocardioides | g__Rhodococcus | 0.903535 |
| g__Nocardioides | g__Escherichia-Shigella | 0.832139 |
| g__Nocardioides | g__Novosphingobium | 0.857267 |
| g__Nocardioides | g__unclassified_f__Enterobacteriaceae | 0.666987 |
| g__Nocardioides | g__unclassified_f__Alcaligenaceae | 0.841134 |
| g__Subdoligranulum | g__Lactobacillus | 1 |
| g__Subdoligranulum | g__norank_f__Bacteroidales_S24-7_group | 1 |
| g__Subdoligranulum | g__Faecalibacterium | 1 |
| g__Ralstonia | g__Rhizobium | 0.881318 |
| g__Ralstonia | g__Sphingomonas | 0.864529 |
| g__Ralstonia | g__Bacillus | 0.609744 |
| g__Ralstonia | g__Methylotenera | 0.743401 |
| g__Ralstonia | g__Phenylobacterium | 0.908297 |
| g__Ralstonia | g__Roseateles | 0.919716 |
| g__Ralstonia | g__Arthrobacter | 0.831508 |
| g__Ralstonia | g__Streptomyces | 0.803923 |
| g__Ralstonia | g__Rhodococcus | 0.853761 |
| g__Ralstonia | g__Escherichia-Shigella | 0.643334 |
| g__Ralstonia | g__Novosphingobium | 0.831547 |
| g__Ralstonia | g__unclassified_f__Alcaligenaceae | 0.680992 |
| g__Prevotella_9 | g__Rhizobium | 0.6046 |
| g__Prevotella_9 | g__Sphingomonas | 0.722211 |
| g__Prevotella_9 | g__Phenylobacterium | 0.64464 |
| g__Prevotella_9 | g__Roseateles | 0.636253 |
| g__Prevotella_9 | g__Streptomyces | 0.718243 |
| g__Prevotella_9 | g__Rhodococcus | 0.619938 |
| g__Prevotella_9 | g__Novosphingobium | 0.694181 |
| g__Rhizobium | g__Sphingomonas | 0.822295 |
| g__Rhizobium | g__Bacillus | 0.70951 |
| g__Rhizobium | g__Methylotenera | 0.752466 |
| g__Rhizobium | g__Phenylobacterium | 0.878623 |
| g__Rhizobium | g__Roseateles | 0.915285 |
| g__Rhizobium | g__Arthrobacter | 0.740577 |
| g__Rhizobium | g__Streptomyces | 0.833068 |
| g__Rhizobium | g__Rhodococcus | 0.896832 |
| g__Rhizobium | g__Escherichia-Shigella | 0.631039 |
| g__Rhizobium | g__Novosphingobium | 0.902533 |
| g__Rhizobium | g__unclassified_f__Alcaligenaceae | 0.741373 |
| g__Sphingomonas | g__Bacillus | 0.654023 |
| g__Sphingomonas | g__Methylotenera | 0.797046 |
| g__Sphingomonas | g__Phenylobacterium | 0.824091 |
| g__Sphingomonas | g__Roseateles | 0.870528 |
| g__Sphingomonas | g__Arthrobacter | 0.830027 |
| g__Sphingomonas | g__Streptomyces | 0.914591 |
| g__Sphingomonas | g__Rhodococcus | 0.932709 |
| g__Sphingomonas | g__Escherichia-Shigella | 0.726135 |
| g__Sphingomonas | g__Sinorhizobium | 0.604255 |
| g__Sphingomonas | g__Novosphingobium | 0.919512 |
| g__Sphingomonas | g__unclassified_f__Alcaligenaceae | 0.833691 |
| g__Bacillus | g__Enterobacter | 0.611879 |
| g__Bacillus | g__Phenylobacterium | 0.602702 |
| g__Bacillus | g__Roseateles | 0.711067 |
| g__Bacillus | g__Rhodococcus | 0.687753 |
| g__Bacillus | g__Escherichia-Shigella | 0.896233 |
| g__Bacillus | g__Novosphingobium | 0.627136 |
| g__Bacillus | g__unclassified_f__Enterobacteriaceae | 0.773237 |
| g__Bacillus | g__unclassified_f__Alcaligenaceae | 0.638442 |
| g__Enterobacter | g__Escherichia-Shigella | 0.758531 |
| g__Enterobacter | g__unclassified_f__Enterobacteriaceae | 0.820815 |
| g__Methylotenera | g__Phenylobacterium | 0.772803 |
| g__Methylotenera | g__Roseateles | 0.806028 |
| g__Methylotenera | g__Arthrobacter | 0.722157 |
| g__Methylotenera | g__Streptomyces | 0.740245 |
| g__Methylotenera | g__Rhodococcus | 0.772129 |
| g__Methylotenera | g__Novosphingobium | 0.862126 |
| g__Methylotenera | g__unclassified_f__Alcaligenaceae | 0.849057 |
| g__Lactobacillus | g__norank_f__Bacteroidales_S24-7_group | 1 |
| g__Lactobacillus | g__Faecalibacterium | 1 |
| g__Phenylobacterium | g__Roseateles | 0.952192 |
| g__Phenylobacterium | g__Arthrobacter | 0.759964 |
| g__Phenylobacterium | g__Streptomyces | 0.714571 |
| g__Phenylobacterium | g__Rhodococcus | 0.8304 |
| g__Phenylobacterium | g__Escherichia-Shigella | 0.715742 |
| g__Phenylobacterium | g__Novosphingobium | 0.837551 |
| g__Phenylobacterium | g__unclassified_f__Alcaligenaceae | 0.767257 |
| g__Roseateles | g__Arthrobacter | 0.74647 |
| g__Roseateles | g__Streptomyces | 0.764456 |
| g__Roseateles | g__Rhodococcus | 0.902256 |
| g__Roseateles | g__Escherichia-Shigella | 0.776526 |
| g__Roseateles | g__Novosphingobium | 0.853149 |
| g__Roseateles | g__unclassified_f__Enterobacteriaceae | 0.609023 |
| g__Roseateles | g__unclassified_f__Alcaligenaceae | 0.866292 |
| g__norank_f__Bacteroidales_S24-7_group | g__Faecalibacterium | 1 |
| g__Arthrobacter | g__Streptomyces | 0.781993 |
| g__Arthrobacter | g__Rhodococcus | 0.793865 |
| g__Arthrobacter | g__Escherichia-Shigella | 0.616389 |
| g__Arthrobacter | g__Novosphingobium | 0.807449 |
| g__Arthrobacter | g__unclassified_f__Alcaligenaceae | 0.605424 |
| g__Streptomyces | g__Rhodococcus | 0.841267 |
| g__Streptomyces | g__Novosphingobium | 0.937402 |
| g__Streptomyces | g__unclassified_f__Alcaligenaceae | 0.657792 |
| g__Rhodococcus | g__Bradyrhizobium | -0.623598 |
| g__Rhodococcus | g__Escherichia-Shigella | 0.698873 |
| g__Rhodococcus | g__Novosphingobium | 0.860504 |
| g__Rhodococcus | g__unclassified_f__Alcaligenaceae | 0.862525 |
| g__Bradyrhizobium | g__Sinorhizobium | -0.847637 |
| g__Bradyrhizobium | g__unclassified_f__Alcaligenaceae | -0.770069 |
| g__Escherichia-Shigella | g__Novosphingobium | 0.611885 |
| g__Escherichia-Shigella | g__unclassified_f__Enterobacteriaceae | 0.836922 |
| g__Escherichia-Shigella | g__unclassified_f__Alcaligenaceae | 0.717479 |
| g__Sinorhizobium | g__unclassified_f__Alcaligenaceae | 0.713199 |
| g__Novosphingobium | g__unclassified_f__Alcaligenaceae | 0.773702 |
| g__unclassified_f__Enterobacteriaceae | g__unclassified_f__Alcaligenaceae | 0.617704 |

**Table S12** The sequence number of pre treatment in treated-soil samples

| **Compartment** | **Soil type** | **Seplicates** | **Seq_num** |
| --- | --- | --- | --- |
| Rhizosphere | HAc | 1 | 41709 |
| Rhizosphere | HAc | 2 | 44978 |
| Rhizosphere | HAc | 3 | 36075 |
| Rhizosphere | HAc | 4 | 35432 |
| Rhizosphere | HNe | 1 | 37818 |
| Rhizosphere | HNe | 2 | 44588 |
| Rhizosphere | HNe | 3 | 33566 |
| Rhizosphere | HNe | 4 | 32717 |
| Rhizosphere | HAl | 1 | 35995 |
| Rhizosphere | HAl | 2 | 40069 |
| Rhizosphere | HAl | 3 | 30585 |
| Rhizosphere | HAl | 4 | 31582 |
| Rhizosphere | Ac/Al | 1 | 54519 |
| Rhizosphere | Ac/Al | 2 | 56151 |
| Rhizosphere | Ac/Al | 3 | 76573 |
| Rhizosphere | Ac/Al | 4 | 71419 |
| Rhizosphere | Ne/Al | 1 | 52727 |
| Rhizosphere | Ne/Al | 2 | 55853 |
| Rhizosphere | Ne/Al | 3 | 94693 |
| Rhizosphere | Ne/Al | 4 | 95823 |
| Rhizosphere | Ac8 | 1 | 55447 |
| Rhizosphere | Ac8 | 2 | 54140 |
| Rhizosphere | Ac8 | 3 | 69607 |
| Rhizosphere | Ac8 | 4 | 62488 |
| Rhizosphere | Ne8 | 1 | 59252 |
| Rhizosphere | Ne8 | 2 | 57314 |
| Rhizosphere | Ne8 | 3 | 53513 |
| Rhizosphere | Ne8 | 4 | 44761 |
| Nodule | HAc | 1 | 32459 |
| Nodule | HAc | 2 | 44883 |
| Nodule | HAc | 3 | 42229 |
| Nodule | HAc | 4 | 33027 |
| Nodule | HNe | 1 | 40642 |
| Nodule | HNe | 2 | 31831 |
| Nodule | HNe | 3 | 41213 |
| Nodule | HNe | 4 | 41835 |
| Nodule | HAl | 1 | 37672 |
| Nodule | HAl | 2 | 41822 |
| Nodule | HAl | 3 | 32405 |
| Nodule | HAl | 4 | 34496 |
| Nodule | Ac/Al | 1 | 49510 |
| Nodule | Ac/Al | 2 | 37441 |
| Nodule | Ac/Al | 3 | 57797 |
| Nodule | Ac/Al | 4 | 50476 |
| Nodule | Ne/Al | 1 | 49006 |
| Nodule | Ne/Al | 2 | 38916 |
| Nodule | Ne/Al | 3 | 65828 |
| Nodule | Ne/Al | 4 | 70430 |
| Nodule | Ac8 | 1 | 31440 |
| Nodule | Ac8 | 2 | 45205 |
| Nodule | Ac8 | 3 | 46656 |
| Nodule | Ac8 | 4 | 89833 |
| Nodule | Ne8 | 1 | 40033 |
| Nodule | Ne8 | 2 | 39975 |
| Nodule | Ne8 | 3 | 34385 |
| Nodule | Ne8 | 4 | 42170 |

**Table S13** General features of the high-throughput sequencing results in treated-soil samples

| **Compartment** | **Number of sequence** | **Number of OTU97** | **Number of genera** | **Number of family** | **Number of order** | **Number of class** | **Number of phylum** | **Coverage** | **Richness** | | **Diversity** | |
| --- | --- | --- | --- | --- | --- | --- | --- | --- | --- | --- | --- | --- |
|  |  |  |  |  |  |  |  |  | **Chao1** | **p.value** | **Shannon** | **p.value** |
| Rhizosphere | 52121 | 2546 | 729 | 368 | 195 | 80 | 32 | 0.988 | 956.8 | 2.28E-05 | 4.31 | 1.72E-09 |
| Nodule | 44415 | 1396 | 529 | 249 | 113 | 47 | 23 | 0.996 | 296.97 |  | 1.31 |  |

**Table S14** Comparisons of Ensifer relativeabundance between different soil type in rhizosphere sample

| Compartment | Soil type 1 | Soil type 2 | Soil type 1 Mean | Soil type 2 Mean | p.value |
| --- | --- | --- | --- | --- | --- |
| Nodule | Ac | HAc | 0.0813 | 0 | 0.28 |
|  | Ac | Ac/Al | 0.0813 | 86.56 | 0.001101 |
|  | Ac | Ac8 | 0.0813 | 0.2187 | 0.363 |
|  | Ne | HNe | 0.0077 | 54.64 | 0.0405 |
|  | Ne | Ne/Al | 0.0077 | 4.487 | 0.3699 |
|  | Ne | Ne8 | 0.0077 | 0.4288 | 0.1774 |
|  | Al | HAl | 98.56 | 99.17 | 0.5444 |

**Table S15** PERMANOVA analysis results using Bray-Curtis as a distance metric for three cluster samples

| **Characteristics** | **SumsOfSqs** | **MeanSqs** | **F.Model** | **R2** | **P.value** | **P.adjust** |
| --- | --- | --- | --- | --- | --- | --- |
| Cluster | 4.65847 | 2.32923 | 15.34324 | 0.45336 | 0.001 | 0.001 |

**Table S16** Identification of Bacillus isolates in alkaline soil

| **Isolates** | **Closest related species** | **Similarity (%)** |
| --- | --- | --- |
|  | **(GenBank Acc. No.)** |  |
| B-9 | *Bacillus wiedmannii* FSL W8-0169 (LOBC01000053) | 99.93% |
| B-11 | *Bacillus cereus* ATCC14579 (AE016877) | 99.79% |
| B-13 | *Bacillus albus* N35-10-2 (MAOE01000087) | 99.91% |

**Table S17** Average nucleotide identity (ANI) of the strains by JSpeciesWS

| **Isolates** | **B-9** | **B-11** | **B-13** | **Bacillus cereus ATCC 14579 [T]** | **Bacillus albus N35-10-2 [T]** | **Bacillus anthracis HYU01** | **Bacillus mobilis 0711P9-1 [T]** | **Bacillus mycoides ATCC 6462 [T]** | **Bacillus thuringiensis CTC** | **Bacillus wiedmannii FSL W8-0169 [T]** |
| --- | --- | --- | --- | --- | --- | --- | --- | --- | --- | --- |
| **B-9** | * | 91.42 | **95.82** | 91.26 | **95.61** | 93.38 | 94.05 | 89.64 | 93.44 | 94.09 |
| **B-11** | 91.05 | * | 91.23 | **97.62** | 91.27 | 91.11 | 90.58 | 89.11 | 91.54 | 90.98 |
| **B-13** | **95.52** | 91.26 | * | 90.99 | **98.45** | 92.99 | 93.37 | 89.2 | 92.88 | 93.33 |
| **Bacillus cereus ATCC 14579 [T]** | 91.01 | **97.98** | 91.16 | * | 91.01 | 91.2 | 90.66 | 88.99 | 91.55 | 90.95 |
| **Bacillus albus N35-10-2 [T]** | **95.06** | 90.95 | **98.26** | 90.59 | * | 92.67 | 92.97 | 88.64 | 92.54 | 92.93 |
| **Bacillus anthracis HYU01** | 93.21 | 91.24 | 93.16 | 91.11 | 93.01 | * | 92.35 | 88.97 | 93.67 | 92.64 |
| **Bacillus mobilis 0711P9-1 [T]** | 93.61 | 90.35 | 93.17 | 90.37 | 93.05 | 92.16 | * | 89 | 92.73 | 93.87 |
| **Bacillus mycoides ATCC 6462 [T]** | 89.16 | 89.04 | 88.96 | 88.76 | 88.92 | 88.7 | 89.04 | * | 89 | 89.6 |
| **Bacillus thuringiensis CTC** | 93.35 | 91.67 | 93.01 | 91.49 | 92.99 | 93.7 | 92.94 | 89.23 | * | 93.31 |
| **Bacillus wiedmannii FSL W8-0169 [T]** | 93.99 | 91.09 | 93.51 | 90.89 | 93.33 | 92.62 | 94.03 | 89.75 | 93.23 | * |

Richter M, Rosselló-Móra R, Glöckner FO, and Peplies J (2015) JSpeciesWS: a web server for prokaryotic species circumscription based on pairwise genome comparison. Bioinformatics. 2015 Nov 16. pii: btv681
